# Manganese is critical for antitumor immune responses via cGAS-STING and improves the efficacy of clinical immunotherapy

**DOI:** 10.1038/s41422-020-00395-4

**Published:** 2020-08-24

**Authors:** Mengze Lv, Meixia Chen, Rui Zhang, Wen Zhang, Chenguang Wang, Yan Zhang, Xiaoming Wei, Yukun Guan, Jiejie Liu, Kaichao Feng, Miao Jing, Xurui Wang, Yun-Cai Liu, Qian Mei, Weidong Han, Zhengfan Jiang

**Affiliations:** 1 Key Laboratory of Cell Proliferation and Differentiation of the Ministry of Education, School of Life Sciences, Peking University, Beijing, 100871 China; 2 Peking-Tsinghua Center for Life Sciences, Peking University, Beijing, 100871 China; 3 Department of Bio-therapeutic, the First Medical Center, Chinese PLA General Hospital, Beijing, 100853 China; 4 Institute for Immunology, Peking-Tsinghua Center for Life Sciences, School of Medicine, Tsinghua University, Beijing, 100084 China; 5 Present Address: Jill Roberts Institute for Research in Inflammatory Bowel Disease (JRI), Weill Cornell Medicine, Cornell University, New York, NY USA; 6 Present Address: Experimental Immunology Branch, National Cancer Institute, National Institutes of Health, Bethesda, MD USA

**Keywords:** Pattern recognition receptors, Immunosurveillance

## Abstract

CD8^+^ T cell-mediated cancer clearance is often suppressed by the interaction between inhibitory molecules like PD-1 and PD-L1, an interaction acts like brakes to prevent T cell overreaction under normal conditions but is exploited by tumor cells to escape the immune surveillance. Immune checkpoint inhibitors have revolutionized cancer therapeutics by removing such brakes. Unfortunately, only a minority of cancer patients respond to immunotherapies presumably due to inadequate immunity. Antitumor immunity depends on the activation of the cGAS-STING pathway, as STING-deficient mice fail to stimulate tumor-infiltrating dendritic cells (DCs) to activate CD8^+^ T cells. STING agonists also enhance natural killer (NK) cells to mediate the clearance of CD8^+^ T cell-resistant tumors. Therefore STING agonists have been intensively sought after. We previously discovered that manganese (Mn) is indispensable for the host defense against cytosolic dsDNA by activating cGAS-STING. Here we report that Mn is also essential in innate immune sensing of tumors and enhances adaptive immune responses against tumors. Mn-insufficient mice had significantly enhanced tumor growth and metastasis, with greatly reduced tumor-infiltrating CD8^+^ T cells. Mechanically, Mn^2+^ promoted DC and macrophage maturation and tumor-specific antigen presentation, augmented CD8^+^ T cell differentiation, activation and NK cell activation, and increased memory CD8^+^ T cells. Combining Mn^2+^ with immune checkpoint inhibition synergistically boosted antitumor efficacies and reduced the anti-PD-1 antibody dosage required in mice. Importantly, a completed phase 1 clinical trial with the combined regimen of Mn^2+^ and anti-PD-1 antibody showed promising efficacy, exhibiting type I IFN induction, manageable safety and revived responses to immunotherapy in most patients with advanced metastatic solid tumors. We propose that this combination strategy warrants further clinical translation.

## Introduction

The immune system recognizes and kills tumor cells, in which the innate immunity is mainly responsible for the detection of tumor cells and the activation of adaptive immunity. However, clearance of tumor cells is often introverted by the interaction between the inhibitory checkpoint molecules like the programmed cell death protein-1 (PD-1)^1,2^ and its ligand such as PD-L1,^3^ an interaction like a brake to prevent T cell overreaction under normal conditions. PD-1 is highly expressed on exhausted T cells, especially on tumor infiltrating lymphocytes (TILs), whereas PD-L1 is highly expressed on and thus exploited by many tumor cells to escape the immune surveillance. Immune checkpoint blockades (ICBs) such as PD-1/PD-L1 or CTLA-4 blocking antibodies have revolutionized cancer therapeutics by removing such inhibitory brakes,^4,5^ the effectiveness of which depends on the recognition of tumor-specific antigens to generate and activate tumor-specific CD8^+^ T cells (also known as cytotoxic T cells, CTLs). In addition, NK cells have been found to mediate the clearance of CD8^+^ T cell-resistant tumors.^6^ Unfortunately, only about 20% of cancer patients respond to immunotherapies presumably due to inadequate immune activation.^5,7,8^

Type I interferons (IFNs) activate both innate and adaptive immunity to promote direct (tumor cell inhibition) and indirect antitumor effects by stimulating the maturation and activation of DCs and macrophages for antigen presentation, increasing the production of granzymes and perforin by both CTLs and NK cells,^6,9^ and enhancing the proportion of memory T cells.^10–12^ Recent works demonstrated that various antitumor therapies depend on the activation of the cGAS-STING pathway,^13–15^ as tumor-derived DNA was found in the cytosol of the tumor-infiltrating DCs and was able to activate this pathway, promoting tumor-specific antigen presentation and CTL activation.^16,17^ Importantly, STING-deficient mice failed to stimulate CD8^+^ T cells in various antitumor therapies.^13,14,18–20^ Consistently, combination of the STING ligand cGAMP and PD-L1 antibody displayed stronger antitumor effects.^17,18^ Interestingly, it has been reported that tumor-derived cGAMP triggers a STING-mediated interferon response in non-tumor cells to activate NK cells^9^ that mediate the clearance of CD8^+^ T cell-resistant tumors in response to STING agonists,^6^ indicating that STING activation promoted antitumor responses to both CD8^+^ T cell-sensitive and CD8^+^ T cell-resistant tumors. Therefore, STING agonists have been studied intensively in recent years.^21,22^

Manganese (Mn) is a nutritional inorganic trace element required for a variety of physiological processes including development, antioxidant defenses, reproduction and neuronal function.^23,24^ Mn (Mn^2+^ in general cases) incorporates into a number of metalloenzymes such as Mn superoxide dismutase (SOD, Mn^3+^ or Mn^2+^ in this case), glutamine synthetase (GS), pyruvate carboxylase, and arginase,^25^ where it plays a critical role in controlling these enzymes. However, its function in immunity is largely unknown. Previously we found that Mn^2+^ was required for the host defense against DNA virus by increasing the sensitivity of the DNA sensor cGAS and its downstream adaptor protein STING. Mn^2+^ was released from mitochondria and Golgi apparatus upon virus infection and accumulated in the cytosol where it bound to cGAS, enhancing the sensitivity of cGAS to double-stranded DNA (dsDNA) and its enzymatic activity. Mn^2+^ also enhanced cGAMP-STING binding affinity. Importantly, Mn^2+^ itself was a potent cGAS activator, inducing cells to produce type I IFNs and cytokines in the absence of any infection.^26^

In this work we discovered that Mn^2+^ is also essential in innate immune sensing of tumors as Mn-insufficient mice had significantly enhanced tumor growth and metastasis, with greatly reduced tumor-infiltrating CD8^+^ T cells. We demonstrated that Mn^2+^ greatly promoted DC and macrophage maturation and antigen presentation, augmented CD8^+^ T cell and NK cell activation, and increased number of CD44^hi^CD8^+^ T cells in a cGAS-STING-dependent way. Mn^2+^ administration thus significantly boosted antitumor immunotherapies in various mouse models. More importantly, a completed phase 1 clinical trial in patients with advanced metastatic solid tumors provided encouraging evidences supporting the manageable safety profile and promised antitumor effects of Mn^2+^ in patients. Importantly, clinical observations suggested that Mn^2+^ administration triggered type I IFN induction and manageable suspected cytokine-release syndrome, augmenting and/or reviving their responses to immunotherapy in patients. This phase 1 study therefore warranted further clinical applications.

## Results

### Mn is essential for antitumor immune responses

**Fig. 1:**
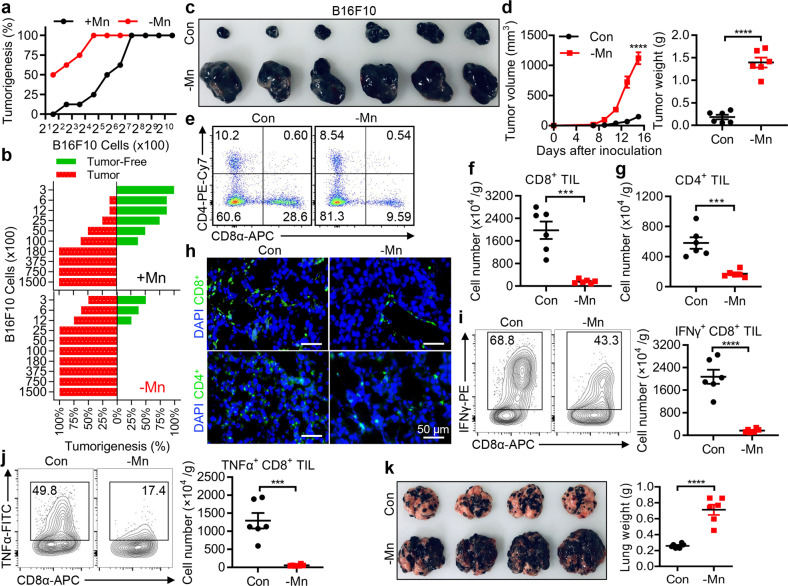
Mn is essential for immune responses against tumors. **a, b** Wild-type (WT) Mn-insufficient (–Mn) and control (+Mn) mice were inoculated with the indicated numbers of B16F10 cells subcutaneously (*n* = 8). Tumorigenesis was monitored every other day for 90 days (**a**). Tumor volume above 50 mm^3^ was defined as tumorigenesis, tumor volume below 50 mm^3^ after 90 days was recorded as tumor-free (**b**). **c, d** Representative images of tumors (**c**), tumor sizes and tumor weights (**d**) in WT control (Con) and Mn-insufficient mice (–Mn) (*n* = 6 per group) after subcutaneous (s.c.) inoculation of 1 × 10^5^ B16F10 cells. **e** Representative FACS data of the frequency of tumor infiltrating CD8^+^ T cells of mice as in (**c**). **f, g** Quantification of tumor infiltrating CD8^+^ T cells (**f**) or CD4^+^ T cells (**g**) of the mice as in (**a**). **h** B16F10 tumors from (**c**) were stained with anti-CD8-FITC or anti-CD4-FITC and counterstained with DAPI. Scale bar, 50 μm. **i, j** Representative FACS data and quantification of tumor infiltrating IFNγ^+^CD8^+^ T cells (**i**) and TNFα^+^CD8^+^ T cells (**j**) of mice as in (**c**). **k** Representative images (left) and quantification of lung weights (right) in control (Con) and Mn-insufficient mice (–Mn) (*n* = 6 per group) at day 21 after intravenous (i.v.) injection of 1 × 10^5^ B16F10 cells. Data represent analyses of the indicated *n* mice per group, means ± SEM. Data are representative of three independent experiments. ***P < 0.001; ****P < 0.0001.

Since we previously found that Mn was critically involved in innate immune sensing of cytosolic dsDNA to activate the cGAS-STING pathway, which is important for exerting antitumor immune responses, we hypothesized that Mn would also play roles in innate immune responses against tumor cells. To test this, Mn-insufficient wild-type (WT) C57BL/6 mice were first generated,^26^ followed by mouse melanoma cell B16F10 inoculation into the groin subcutaneously. 100% of tumor occurrence was observed in Mn-insufficient mice inoculated with a minimum of 2.5 × 10^3^ tumor cells, while seven times more tumor cells (1.8 × 10^4^) were required to induce tumor occurrence in the control WT mice (Fig. 1a, b). Further, we confirmed that Mn-insufficient mice were much more vulnerable to B16F10 tumor invasion compared to the control mice, as indicated by the greatly increased tumor size and weight (Fig. 1c, d) with significantly reduced tumor-infiltrating CD8^+^ and CD4^+^ T cells (TILs) in tumors (Fig. 1e–h). Deprivation of Mn did not affect the percentage of CD4^+^ and CD8^+^ T cells, suggesting that T cell development is normal in Mn-insufficient mice (Supplementary information, Fig. S1a). Consistently, much reduced IFNγ-producing (Fig. 1i) and TNFα-producing (Fig. 1j) CD8^+^ TILs were found in tumors from Mn-insufficient mice. Strikingly, intravenously inoculated B16F10 cells in Mn-insufficient mice induced much stronger lung metastasis, compared to the control Mn-sufficient mice (Fig. 1k). These results demonstrated Mn is critical in maintaining the host antitumor immune responses and strongly suggested that Mn is important for preventing tumorigenesis under physiological conditions.

### Mn^2+^ promotes antitumor immune responses

Since Mn^2+^ is an activator of the cGAS-STING pathway, we went to test if Mn^2+^ would promote antitumor immune responses in vivo. We subcutaneously inoculated C57BL/6 mice with B16F10 or mouse colon adenocarcinoma cell MC38 into the groin, followed by Mn^2+^ administration intranasally or intravenously, we found that Mn^2+^ treatment by both routes significantly reduced tumor burdens (Supplementary information, Fig. S1b–e). Similar results were obtained using the Lewis Lung Carcinoma (LLC) and E.G7 tumor models (Supplementary information, Fig. S1f, g). Specifically, LLC tumor-bearing mice treated with Mn^2+^ survived significantly longer than the control mice (Supplementary information, Fig. S1h). Next, B16F10 cells were inoculated intravenously to induce lung metastasis. Intranasal Mn^2+^ administration resulted in fewer tumor nodules in lungs (Supplementary information, Fig. S1i, j), suggesting a robust systemic antitumor response. No significant cell death was observed either when mouse fibroblast cells or these tumor cells were directly treated by Mn^2+^ at concentrations used in this study (Supplementary information, Fig. S1k). Importantly, intratumoral Mn^2+^ injection effectively inhibited tumor growth as more than 50% of treated mice (13/23) were tumor-free (Supplementary information, Fig. S2a). Moreover, intratumoral Mn^2+^ injection of tumors on one side induced suppression of the other side, non-injected tumors with significantly prolonged survival (Supplementary information, Fig. S2b, c), again suggesting that Mn^2+^ administration induces a systemic antitumor response. These results also indicated that Mn^2+^ is a potent chemotherapeutic agent by itself. Further, there was no significant difference on body weight, survival and gross organ anatomy between the control and Mn^2+^-treated mice during the experiment (Supplementary information, Fig. S2d–l). Collectively, these results suggested that Mn effectively  promoted host antitumor immune responses.

### Mn^2+^ stimulates CD8^+^ T cell and NK cell activation

**Fig. 2:**
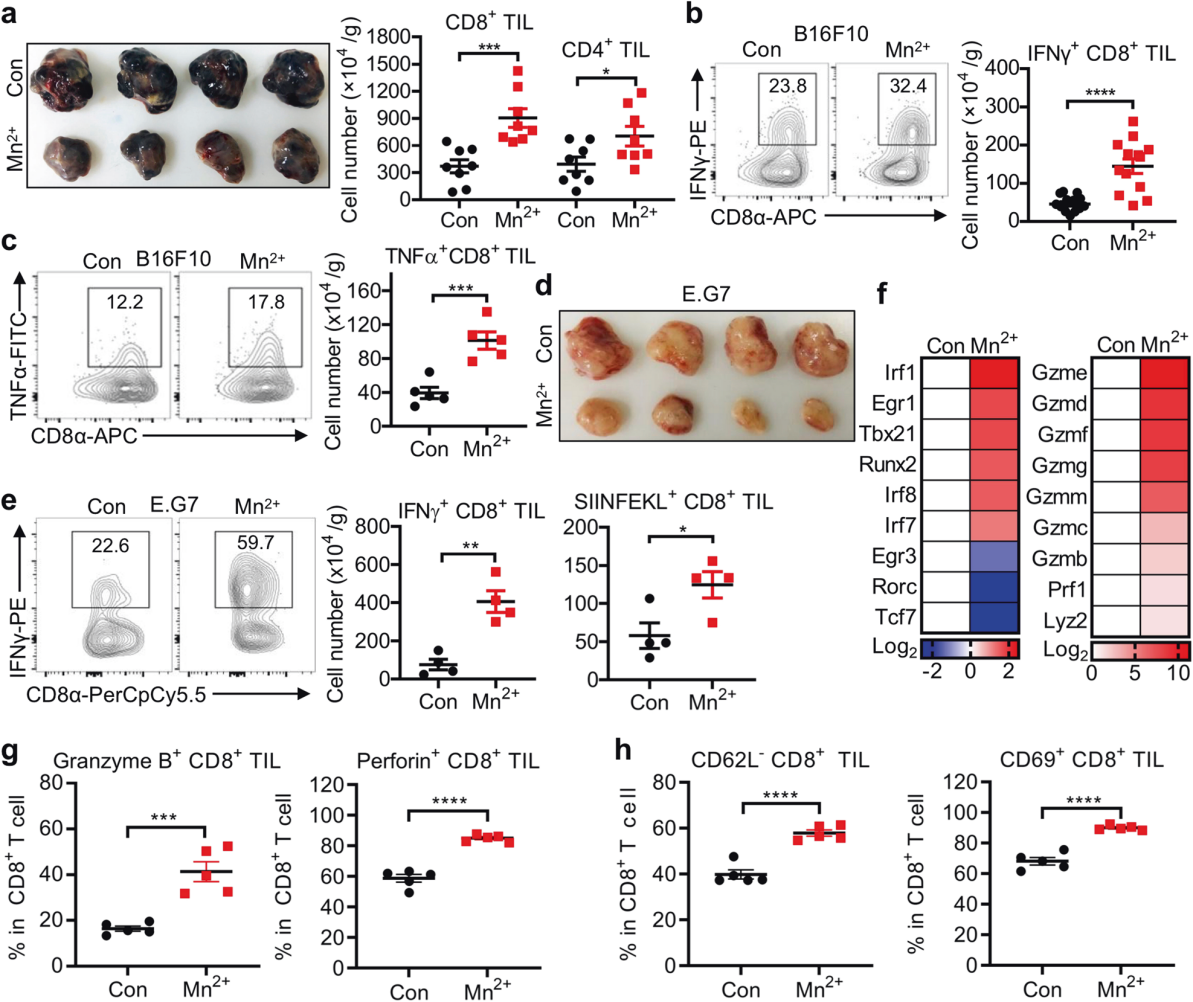
Mn^2+^ stimulates CD8^+^ T cell and NK cell activation. **a** Representative image of tumors in the WT mice (*n* = 8 per group) treated with saline or 5 mg/kg MnCl_2_ intranasally (i.n.) at day 14 after subcutaneous inoculation of 5 × 10^5^ B16F10 cells (left) and quantification of tumor infiltrating CD8^+^ T cells or CD4^+^ T cells (right). **b, c** Representative FACS data of frequency (left) and quantification (right) of tumor infiltrating IFNγ^+^CD8^+^ T cells (**b**, *n* = 13 per group) or TNFα^+^CD8^+^ T cells (**c**, *n* = 5 per group) in the WT mice treated with saline or 5 mg/kg MnCl_2_ i.n. at day 15 after subcutaneous inoculation of 5 × 10^5^ B16F10 cells. **d, e** Images of tumors (**d**), representative FACS data of frequency (**e**, left) and quantification (**e**, right) of tumor infiltrating IFNγ^+^CD8^+^ T or SIINFEKL^+^CD8^+^ T cells in the WT mice treated with saline or 5 mg/kg MnCl_2_ i.n. at day 17 after subcutaneous inoculation of 1 × 10^6^ E.G7 cells (*n* = 4 per group). **f** Heatmap of selected genes between CD8^+^ TILs from the control and Mn^2+^-treated (i.n.) WT mice. Heatmap was made by calculating log_2_((Mn^2+^ FPKM)/(Con FPKM)) and values of genes in the control group were normalized to zero. **g, h** Quantification of tumor infiltrating Granzyme B^+^CD8^+^ and Perforin^+^CD8^+^ T cells (**g**, *n* = 5 per group) or CD62L^−^CD8^+^ and CD69^+^CD8^+^T cells (**h**, *n* = 5 per group) in the WT mice treated with saline or 5 mg/kg MnCl_2_ i.n. at day 16 after subcutaneous inoculation of 2 × 10^5^ B16F10 cells. Data represent analyses of the indicated *n* mice per group, means ± SEM. Data are representative of three independent experiments. *P < 0.05; **P < 0.01; ***P < 0.001; ****P < 0.0001.

Next we determined which cell subsets conducted the antitumor effect elicited by Mn^2+^. *Rag1*^*−⁄−*^ (Supplementary information, Fig. S3a) or *β2m*^*−⁄−*^ (Supplementary information, Fig. S3b) mice were used to verify that Mn^2+^-triggered antitumor effects depend on CD8^+^ T cells^27^ and NK cells. Since the presence and activity of TILs determine the clinical outcome of immunotherapies, tumors were dissected at the endpoint after inoculation and TILs were analyzed by flow cytometry. Mn^2+^ treatment led to a significantly increased CD8^+^ TILs in B16F10 tumors (Fig. 2a) and in other tumor models (Supplementary information, Fig. S3c). Meanwhile, CD4^+^ TILs were also increased in Mn^2+^-treated mice (Fig. 2a). Consistently, greatly increased IFNγ- (Fig. 2b) and TNFα-producing (Fig. 2c) CD8^+^ TILs were found in tumors from Mn^2+^-treated mice. Further, Mn^2+^-treated E.G7-bearing mice showed obviously reduced tumor size with significantly increased IFNγ-producing CD8^+^ TILs, and specifically more SIINFEKL^+^CD8^+^ TILs (Fig. 2d, e), indicating the enhanced tumor antigen-specific recognition and increased antigen-specific CTLs. Moreover, significantly increased CD107a^+^ and granzyme B^+^ NK cells were observed in tumors after Mn^2+^ administration (Supplementary information, Fig. S3d).

Critically, RNA-sequencing (RNA-seq) analysis of CD8^+^ TILs isolated from the control or Mn^2+^-treated B16-inoculated mice showed that the transcriptional factors IRF1 for IFNγ production and CD8^+^ T cell proliferation,^28,29^ IRF8 and T-bet (Tbx21) for IFNγ production and CD8^+^ T cell differentiation,^30–33^ and Runx2 for memory CD8^+^ T cell development^34^ were all significantly upregulated in TILs of Mn^2+^-treated mice, along with many upregulated effector genes for CD8^+^ T cells, including perforin (Prf1) and various granzymes (Gzm) (Fig. 2f). Interestingly, all granzymes except GzmA were induced by Mn^2+^ treatment, consistent with previous works, including multicenter clinical studies, demonstrating that GzmA is not important in T cell or natural killer (NK) cell-mediated cytotoxicity, instead acts as a pro-inflammatory cytokine contributing to cancer development.^35,36^ The increase of granzyme B and perforin producing CD8^+^ TILs was verified by flow cytometry (Fig. 2g). Moreover, various cytokines (IL1b/18/23a), chemokines (CCL2/8 and CXCL1/2/10/14) and their receptors (CCR1/6 and IL2Rb/12Rb1/21R), known to be important for TIL recruitment and activation,^37,38^ were also highly upregulated in TILs isolated from Mn^2+^-primed mice (Supplementary information, Fig. S3e). In contrast, the expression of Early Growth Response 3 (Egr3) and RAR Related Orphan Receptor C (Rorc), transcription factors suppress both effector CD8^+^ T cell differentiation and IFNγ production,^39–41^ were severely inhibited. The transcription factor 7 (Tcf7) relevant to effector differentiation of most CD8^+^ T cells^42^ was also down-regulated. Significant enhanced CD44^hi^CD8^+^ T cells were detected in Mn^2+^-treated mice (Supplementary information, Fig. S3f–h), indicating more memory and activated CD8^+^ T cells were generated after Mn^2+^ administration,^43^ which could potentially enhance antitumor immune responses. These results were further verified by FACS analysis of tumors revealing significantly increased CD62L^−^CD8^+^ and CD69^+^CD8^+^ T cells (Fig. 2h), demonstrating the enhanced memory T cell proportion and activation. Consistently, the increased CD44 expression on CD8^+^ T cells correlated well with the upregulation of CD44-specific transcription factor Egr1.^44^ These RNA-seq results strongly indicated that Mn^2+^ promotes CD8^+^ T cell differentiation and activation via regulating the expression of various transcription factors. NK cells also play important roles in antitumor immunity, especially at the early stage of tumorigenesis and the clearance of CD8^+^ T cell-resistant tumors.^6,9,45,46^ Since NK cells were not functional in *β2m*^*−⁄−*^ mice,^47^ the involvement of NK cells in Mn^2+^-promoted antitumor responses in these mice could not be determined. So we next tested the effect of Mn^2+^ on NK cells isolated from mouse spleens. Consistent with previous reports demonstrating that Mn^2+^ enhanced NK cell activation,^48,49^ NK cells were highly activated by Mn^2+^ treatment in vitro, as the expression of CD107a and granzyme B was significantly enhanced (Supplementary information, Fig. S3i). Collectively, we concluded that Mn^2+^ promoted antitumor immune responses by activating both CD8^+^ T cells and NK cells for the clearance of CD8^+^ T cell-sensitive and CD8^+^ T cell-resistant tumors.

### Mn^2+^ promotes DC maturation and antigen presentation

**Fig. 3:**
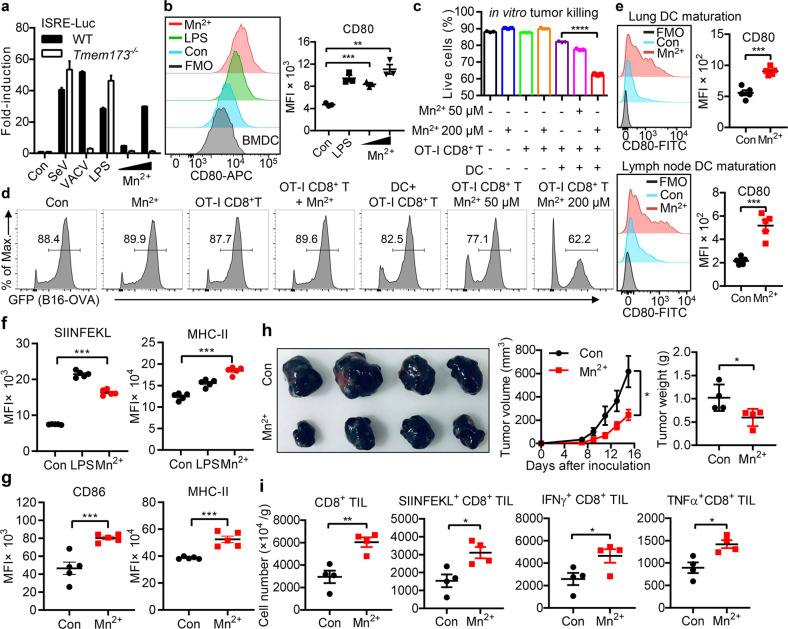
Mn^2+^ promotes DC maturation and antigen presentation. **a** Type I IFN activity in culture media from the WT or *Tmem173*^*−⁄−*^ BMDCs treated with SeV, VACV, LPS or the indicated concentrations (200 μM and 400 μM) of MnCl_2_ for 18 h. **b** Mean fluorescent intensity (MFI) of CD80 in BMDCs treated with LPS (10 ng/mL) or the indicated concentrations (200 μM and 400 μM) of MnCl_2_ for 18 h. FMO: Flow Minus One of CD80. **c, d** CD8^+^ T cells isolated from spleen of OT-I mice (6–8 weeks old) were mixed with BMDCs at 2:1 ratio and incubated with B16F10-OVA-GFP cells with or without the indicated concentrations of MnCl_2_ for 24 h. Viability of tumor cells were analyzed by flow cytometry (FACS). **e** MFI of CD80 in lung DCs (top, MnCl_2_ i.n.) or inguinal lymph node DCs (bottom, MnCl_2_ s.c.) from mice (*n* = 5 per group) treated with 5 mg/kg MnCl_2_ for 18 h. **f** B16F10-OVA cells were co-cultured with BMDCs under indicated treatment for 18 h. Expression of the OVA peptide SIINFEKL–MHC-I molecule complex and co-stimulatory molecule MHC-II on the surface of BMDCs was analyzed by FACS. **g** WT mice were subcutaneously inoculated with 2 × 10^5^ B16F10 cells and treated with saline or 5 mg/kg MnCl_2_ i.p. Mice (*n* = 5 per group) were sacrificed on day 16 and tumors were dissected for FACS analysis. The expression of CD86 and MHC-II on tumor-infiltrating DCs was quantified. **h** Representative images (left) and quantification (right) of tumor sizes and tumor weights in WT mice (*n* = 4 per group) treated with MnCl_2_ i.n. and then inoculated with B16F10 s.c. at day 5 and sacrificed at day 20. Experimental protocol was described in Supplementary information, Fig. S4e. **i** Summary data of CD8^+^ TILs, SIINFEKL^+^CD8^+^ TILs, IFNγ^+^CD8^+^ TILs and TNFα^+^CD8^+^ TILs in tumors from mice in (**h**). Data represent analyses of the indicated *n* mice per group, means ± SEM. Data are representative of three independent experiments. *P < 0.05; **P < 0.01; ***P < 0.001; ****P < 0.0001.

The professional antigen-presenting DCs are activated by type I IFNs and essential for CD8^+^ T cell priming.^11^ We found that Mn^2+^ treatment caused bone marrow-derived DCs (BMDCs) to produce large amounts of type I IFNs (Fig. 3a) and greatly induced DC maturation as LPS did (Fig. 3b; Supplementary information, Fig. S4a). Consistently, Mn^2+^ addition to the in vitro killing assay in a co-culture system containing DCs, CD8^+^ T and B16-OVA cells led to a significantly improved killing of tumor cells (Fig. 3c, d). Importantly, BMDCs and DCs from lungs and lymph nodes isolated from Mn^2+^-treated WT mice displayed much enhanced maturation and elevated capability in antigen presentation (Fig. 3e, f; Supplementary information, Fig. S4b), which were further verified in tumors (Fig. 3g). In agreeing with this, Mn^2+^ potently activated macrophages to produce huge amounts of type I IFNs which contributed to DC maturation (Supplementary information, Fig. S4c and Table S1). Consistently, in vitro and in vivo Mn^2+^ treatment promoted CD86 expression and antigen presentation on macrophages (Supplementary information, Fig. S4d, e). Moreover, intranasal Mn^2+^ administration induced robust TNFα production in alveolar macrophages (Supplementary information, Fig. S4f and Table S1), which also contributed to DC maturation and CD8^+^ T cell activation.^50,51^ Further, Mn^2+^ pretreatment before tumor inoculation significantly increased IFNγ production by CD8^+^ TILs and thus elevated tumor resistance (Fig. 3h, i; Supplementary information, Fig. S4g), indicating a preventative protection by sensitizing the innate immunity. More importantly, Mn^2+^ treatment significantly induced maturation of DCs in peripheral blood mononuclear cells (PBMCs) from various types of cancer patients with a high responsive rate (Supplementary information, Fig. S4h and Table S2).

### The cGAS-STING pathway is required for Mn^2+^-mediated antitumor immune responses

**Fig. 4:**
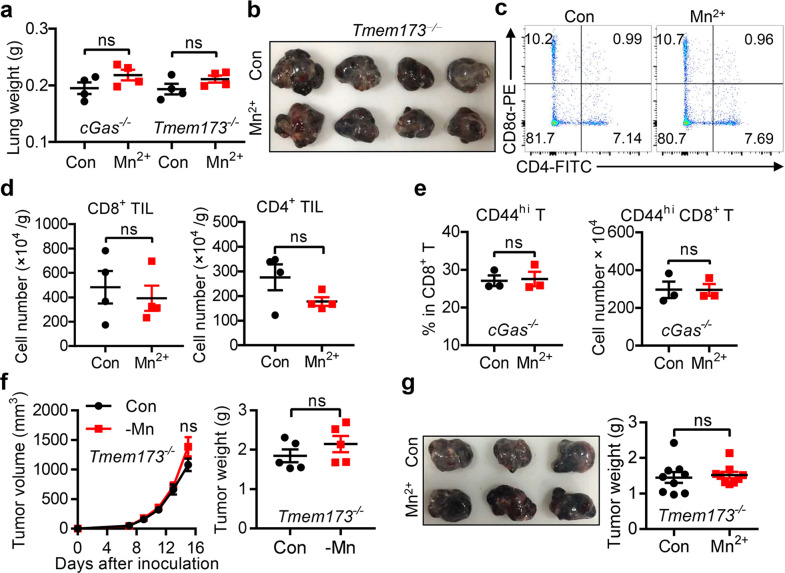
The cGAS-STING pathway is required for Mn^2+^-mediated antitumor immune responses. **a** Quantification of lung weights from *cGas*^*−⁄−*^ mice (top) or *Tmem173*^*−⁄−*^ mice (bottom) with saline or 5 mg/kg MnCl_2_ (i.n., *n* = 4 per group) at day 14 after intravenous injection of 2 × 10^5^ B16F10 cells. **b** Images of tumors in *Tmem173*^*−⁄−*^ mice (*n* = 4 per group) treated with saline or 5 mg/kg MnCl_2_ i.n. at day 14 after subcutaneous inoculation of 5 × 10^5^ B16F10 cells. **c, d** Representative FACS data (**c**) and quantification of tumor infiltrating CD8^+^ T cells or CD4^+^ T cells (**d**) from *Tmem173*^*−⁄−*^ mice as in **b**. **e** Experimental protocol was described in Supplementary information, Fig. S3e: *cGas*^*−⁄−*^ mice (*n* = 3 per group) were given 5 mg/kg MnCl_2_ i.p. at the indicated times and sacrificed at day 9. Frequency (left) and cell number (right) of CD44^hi^CD8^+^ T from splenic cells in *cGas*^*−⁄−*^ mice. **f** Tumor sizes and tumor weights in *Tmem173*^*−⁄−*^ control (Con) and Mn-insufficient mice (–Mn) (*n* = 5 per group) after subcutaneous inoculation of 5 × 10^5^ B16F10 cells. **g** Images of tumors (left), tumor weights (right) in *Tmem173*^*−⁄−*^ mice (*n* = 9 per group) treated with saline or 5 mg/kg MnCl_2_ i.n. at day 14 after subcutaneous inoculation of 5 × 10^5^ B16F10 cells. Data represent analyses of the indicated *n* mice per group, means ± SEM. Data are representative of three independent experiments. ns, not significant, *P* > 0.05.

Using cGAS (*cGas*^*−⁄−*^) and STING (*Tmem173*^*−⁄−*^) deficient mice (Supplementary information, Fig. S5a and Table S3), we confirmed that the cGAS-STING pathway is essential for Mn^2+^-induced type I IFN production (Supplementary information, Fig. S5b) and antitumor effects (Fig. 4a, b). Deletion of these genes in mice caused complete loss of responses to Mn^2+^ treatment with no increased CD8^+^ or CD4^+^ TILs (Fig. 4c, d) or enhanced CD44^hi^CD8^+^ T cell proportion after Mn^2+^ administration (Fig. 4e). Importantly, the growth of tumor cells and antigen-specific CTL activation were not altered in either Mn-insufficient (Fig. 4f) or Mn^2+^-administrated (Fig. 4g) *Tmem173*^*−⁄−*^ mice, confirming the essential role of cGAS-STING in Mn^2+^-mediated host immune responses against tumor cells. We also analyzed the myeloid-derived suppressor cells (MDSCs) in tumors as MDSCs may promote tumor growth and metastasis,^52,53^ however, tumors from Mn^2+^-treated mice and control mice showed no significant difference in MDSC infiltration (data not shown). These data collectively showed that Mn^2+^ promoted DC maturation and triggered a CTL-mediated antitumor effect in a cGAS-STING-dependent way.

### Mn^2+^ shows adjuvant effects on antitumor vaccines

**Fig. 5:**
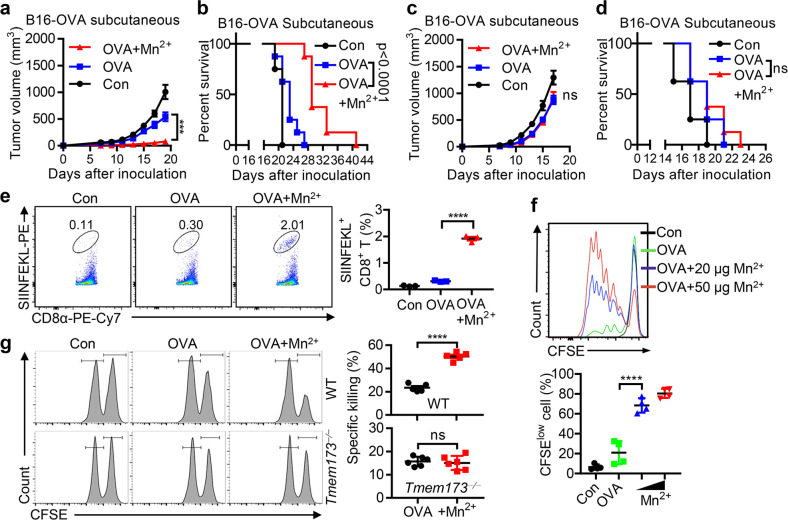
Mn^2+^ shows adjuvant effects on antitumor vaccines. **a, b** Tumor sizes in the WT mice (*n* = 8 per group) pre-immunized with PBS, OVA or OVA plus MnCl_2_ intramuscularly (i.m.) on day 0, 7 or 14, before 5 × 10^5^ B16F0-OVA subcutaneous inoculation on day 21 (**a**). The survival of mice was monitored (**b**). **c, d** Tumor sizes in *Tmem173*^*−⁄−*^ mice (*n* = 8 per group) pre-immunized with PBS, OVA or OVA plus MnCl_2_ i.m. on day 0, 7 or 14, before 5 × 10^5^ B16F0-OVA subcutaneous inoculation on day 21 (**c**). The survival of mice was monitored (**d**). **e** Representative figures and summary data of frequency of SIINFEKL^+^CD8^+^ T cells in the spleen of immunized (PBS, OVA or OVA plus MnCl_2_ i.m. on day 0, 7 or 14) mice on day 21. **f** CD8^+^ T cell proliferation in inguinal lymph nodes from recipient mice at day 3 after immunization with PBS, OVA or OVA plus MnCl_2_ s.c. (*n* = 4 per group). **g** In vivo CTL assay in WT (top panel) or *Tmem173*^*−⁄−*^ mice (bottom panel) 21 d after i.m. (day 0, 7, 14) with OVA (100 μg) with or without MnCl_2_ (20 μg) (*n* = 5 per genotype). Data represent analyses of the indicated *n* mice per group, means ± SEM. Data are representative of three independent experiments. ns, not significant, *P* > 0.05; *** *P* < 0.001; **** *P* < 0.0001.

Given that Mn^2+^ strongly induced type I IFN production and DC maturation, we reasoned that Mn^2+^ could be used as an antitumor adjuvant. By using the B16-OVA tumor model, we intramuscularly immunized C57BL/6 mice with LPS-free chicken ovalbumin protein (OVA) alone or OVA with Mn^2+^ prior to B16-OVA inoculation. We found that Mn^2+^-pre-immunized mice were much more resistant to tumors with significantly suppressed tumor growth (Fig. 5a) and greatly increased survival (Fig. 5b). Importantly, such inhibition disappeared in *Tmem173*^*−⁄−*^ mice (Fig. 5c, d). Consistently, mice immunized with OVA and Mn^2+^ generated many more OVA-specific CTLs (Fig. 5e), promoted CD8^+^ T cell proliferation (Fig. 5f) and enhanced in vivo CTL killing ability (Fig. 5g), demonstrating that Mn^2+^ boosts OVA-specific CD8^+^ T cell proliferation. We thus proposed that Mn^2+^ may be used as an adjuvant in antitumor vaccines similarly to some STING agonists.^54^

### Mn^2+^ boosts antitumor immunotherapy in mice

**Fig. 6:**
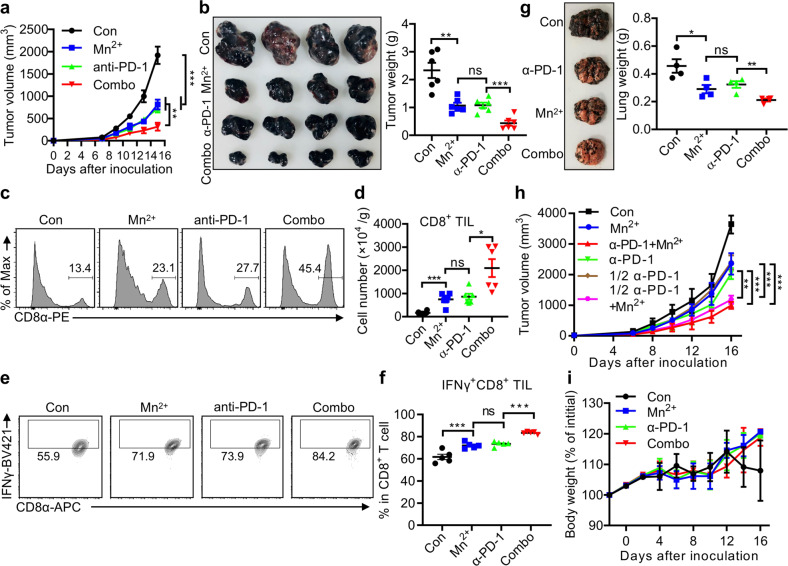
Mn^2+^ boosts antitumor immunotherapy in mice. **a** Tumor sizes of subcutaneous B16F10 implants in mice treated with the isotype antibody (200 μg/mouse i.p.), MnCl_2_ (5 mg/kg i.n.), anti-PD-1 antibody (200 μg/mouse i.p.) or MnCl_2_ plus anti-PD-1 antibody (*n* = 6 per group. Combo, combined treatment with MnCl_2_ and anti-PD-1 antibody). **b** Representative image (left), tumor weights (right) of subcutaneous B16F10 implants in mice as in **a**. **c, d** Representative FACS figures (**c**) and quantification (**d**) of tumor infiltrating CD8^+^ T cells of mice as in **a**. **e, f** Representative FACS figures (**e**) and quantification (**f**) of IFNγ^+^CD8^+^ TILs of mice as in **a**. **g** Representative image (left) and quantification (right) of tumor nodules and lung weights of saline or 5 mg/kg MnCl_2_ treated mice (i.n., *n* = 4 per group) at day 15 after intravenous injection of 2 × 10^5^ B16F10 cells. **h** Tumor sizes of subcutaneous B16F10 implants in mice treated with isotype antibody (200 μg/mouse i.p.), MnCl_2_ (5 mg/kg i.p.), anti-PD-1 (200 μg/mouse i.p.), 1/2 anti-PD-1 (100 μg/mouse i.p.), or 1/2 anti-PD-1 plus MnCl_2_ (*n* = 5 per group). **i** Body weight changes were followed for 16 days after subcutaneous B16F10 implants in mice treated with MnCl_2_ (5 mg/kg i.p.), anti-PD-1 antibody (200 μg/mouse i.p.), or MnCl_2_ plus anti-PD-1 antibody (*n* = 5 per group). Data represent analyses of the indicated *n* mice per group, means ± SEM. Data from cells and mice are representative of three independent experiments. ns, not significant, *P* > 0.05; *P < 0.05; **P < 0.01; ***P < 0.001.

Recent works showed that cGAMP enhanced antitumor effects of immune checkpoint blockades (ICBs) such as PD-1, PD-L1 or CTLA-4 blocking antibodies.^17,18,55^ We proposed that the antitumor immunotherapy may be considered as the vehicle driving the activation of innate immunity, and the cGAS-STING pathway in this case, acts like to press the accelerator or even to start the engine, while ICBs function to release the brake. In fact, B16 melanoma is well recognized as an aggressive tumor with meager immunogenicity and is thus poorly controlled by PD-1 blocking antibodies. Since Mn^2+^ strongly promoted DC maturation as engine-starting and CTL activation like accelerator-pressing, we hypothesized that combined PD-1 blocking antibody with Mn^2+^ would treat B16 melanoma better. We found that this is indeed the case. Intranasal Mn^2+^ administration showed a significant synergic effect on restricting B16 melanoma growth in mice (Fig. 6a, b) with greatly increased CD8^+^ TILs (Fig. 6c, d) and IFNγ^+^CD8^+^ TILs (Fig. 6e, f). Such synergic effect was also found in MC38 tumor (Supplementary information, Fig. S6a–c) and lung metastatic B16F10 melanoma models (Fig. 6g), consistent with clinical observations that checkpoint blockade-caused tumor regression is achieved by the reactivation of CD8^+^ T cells within tumors.^56^ Strikingly, half dose of PD-1 blocking antibodies showed a significantly better effect to inhibit tumor growth when Mn^2+^ was simultaneously administrated (Fig. 6h). Importantly, Mn^2+^ treatment did not reduce the body weight or damage the indicated major organs of mice compared to those treated with anti-PD-1 or combination therapy (Fig. 6i; Supplementary information, Fig. S6d), indicating the safety profile of Mn^2+^ treatment and its translational value. Collectively, these data suggested that Mn^2+^ synergistically improved the efficacy of immunotherapy in mouse models.

### Mn^2+^ augments and revives antitumor immunotherapy in multidrug (immune)-resistant cancer patients

**Fig. 7:**
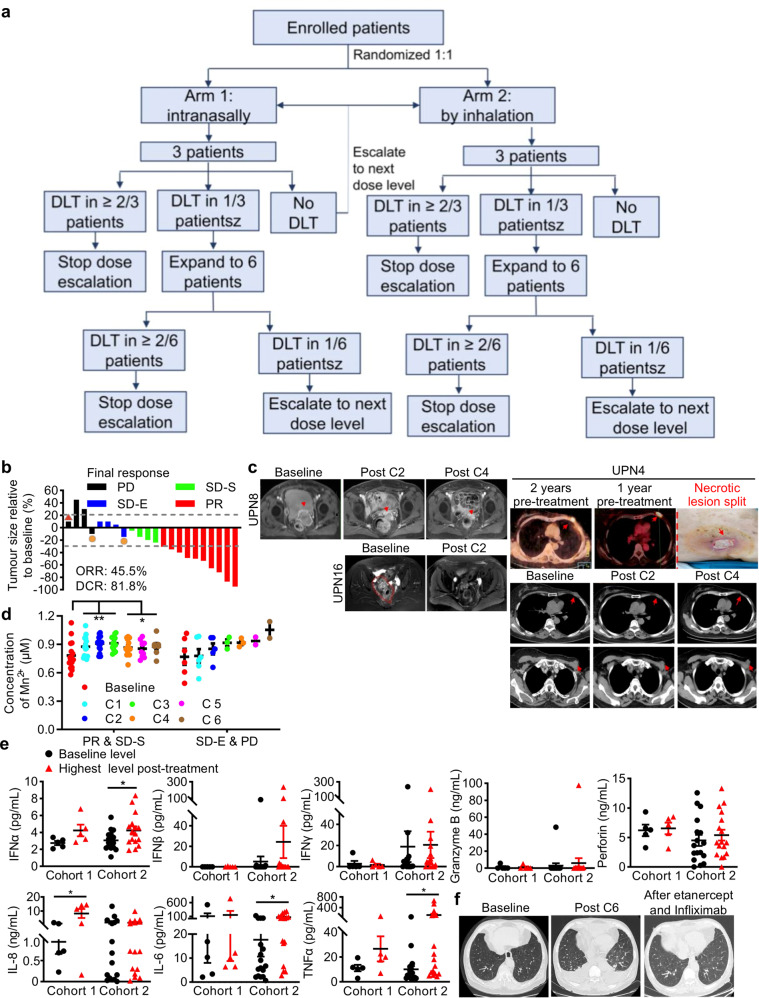
Mn^2+^ augments/revives antitumor immunotherapy in multidrug (immuno)-resistant cancer patients. **a** The graphical abstract of clinical trial design. **b** The best percentage change from baseline in the longest diameters of target lesions. Dashed lines indicated the thresholds regarding progressive disease and partial response per the Response Evaluation Criteria in Solid Tumors (RECIST) v1.1. **c** Representative cases were shown. Patient UPN4 with refractory metastatic breast cancer achieved PR and experienced necrotic lesion splitting away off chest wall after four cycles of the combined therapy. Two patients with platinum-resistant metastatic ovarian cancer achieved PR and impressive partial remission of frozen pelvis following the administration of Mn^2+^. **d** The levels of blood Mn concentration following *n* = C1–C6 cycles of therapy in patients grouped by clinical response. **e** The baseline and highest post-treatment expression level of serum cytokines and effector proteins of patients grouped according to the blood Mn concentration. Cohort 1 included the patients with SD-E and PD; cohort 2 included patients achieving PR and SD-S. **f** Patient UPN1 with refractory colorectal cancer developed acute CRS accompanying hypoxemia resulted from pleural effusion and pulmonary edema, which was resolved by anti-TNFα/TNFαR antibody therapy. DLT, dose limiting toxicity; PR, partial response; SD-S, stable disease with decrease lesion; SD-E, stable disease with enlarged lesion; PD, progressive disease. *P* values were calculated using paired *t*-test (SPSS 26). Data represent means ± SEM; *P < 0.05; **P < 0.01.

Based on the aforementioned data, we conducted a first-in human, open-label, dose-escalation phase 1 clinical trial to assess the safety and preliminary efficacy of Mn^2+^ priming anti-PD-1 antibody plus chemotherapy (ClinicalTrials.gov, NCT03991559). From November 25, 2018 to May 15, 2019, twenty-two patients with advanced metastatic solid tumors were enrolled, and received at least two doses of the combined therapy (Fig. 7a–c; Supplementary information, Tables S2, S4, S5). MnCl_2_ solution was administered intranasally or by inhalation (as described in the Materials and Methods). At data cutoff on April 15, 2020, all patients experienced at least one evaluable post-treatment tumor scan, and showed preliminary favorable clinical efficacy with 45.5% (95% CI, 26.9–65.3) best objective response and 90.9% (95% CI, 72.2–97.5) best disease control rate (Fig. 7b; Supplementary information, Fig. S7a). Consistently, the clinical responses correlated well with the in vitro Mn^2+^ responses by PBMCs isolated from these cancer patients (Supplementary information, Table S2). Critically, all five patients who failed the previous combined treatment of anti-PD-1 antibody and chemotherapy or radiotherapy showed disease control, including three with partial response (PR) and two with stable disease (SD), suggesting that Mn^2+^ revived antitumor immunotherapy in these immune unresponsive patients. Remarkably, slough of necrotic tumor mass from chest wall in one breast cancer patient and largely relief of frozen pelvis in six ovarian cancer patients were observed (Fig. 7c). Concentration of blood free Mn was detected by differential-pulse polarographic determination with a normal range between 0.029 μM and 1.2 μM, which is different with inductively coupled plasma-atomic emission spectroscopy (ICP-AES) recommended by ATSDR (Supplementary information, Data S1). Importantly, post-administration blood Mn levels were elevated but remained within the normal range, and it appeared to predict its antitumor effects (Fig. 7d). Stratification by clinical responses demonstrated that a significant increase of Mn concentration was observed in patients who achieved PR and SD with decreased lesions (Cohort 2) but not in patients with PD and SD with enlarged lesions (Cohort 1), and remained elevated for at least 12 weeks (Fig. 7e). Elevated blood Mn promoted the induction of type I IFNs and some pro-inflammatory cytokines, as statistically significantly increased IFNα, IL-6, IL-8 and TNFα were observed in Cohort 2 (Fig. 7e; Supplementary information, Fig. S7b, Cohort 2), consistent with the immunostimulatory effects of Mn^2+^ in mouse models and human PBMCs.

This phase 1 trial documented a manageable safety profile, any-grade treatment-related adverse events (AEs) occurred in 19 (86%) patients and serious treatment-related AEs (grade 3–4) in nine (41%) patients without any treatment-related death (Supplementary information, Table S6). The grade 1–2 AEs were well tolerated and grade 3–4 AEs resolved with supportive cares. Five patients discontinued treatment because of immune-related pneumonitis (*n* = 2) and chemotherapy-related hematological and gastrointestinal toxicities (*n* = 3). With a median follow-up of 11.8 months, no Mn overdose-related toxicity and accumulation of Mn in the basal ganglia was observed.^57^ Importantly, five patients with extensive metastases in abdominal cavity developed acute suspected local or systemic cytokine-release-syndrome (CRS) with apparent induction of serum type I IFNs and some pro-inflammatory cytokines including IL-6 and TNFα (Supplementary information, Fig. S7c, d), accumulated ascites, diarrhea, ileus and hypoxemia resulted from pleural effusion and pulmonary edema (Fig. 7f), but all resolved by the use of anti-TNFα/TNFαR or anti-IL6R antibody alone. Radiographic data of all 22 patients before and after treatment were all exhibited (Supplementary information, Fig. S8). This phase 1 study thus provided preliminary encouraging evidence supporting the safety profile and antitumor effects of Mn^2+^ in patients with advanced metastatic solid tumors, and now a phase 2 study is ongoing.

## Discussion

Mn is a nutritional inorganic trace element with diverse biological activities. Its immune regulatory effects have never been discovered. In this study we investigated the role of Mn in bridging innate and adaptive immunity for tumor surveillance (Supplementary information, Fig. S7e). We found that Mn played a critical role in innate immune sensing of tumors, as Mn-insufficient mice poorly controlled tumor growth and metastasis. In contrast, tumor growth, metastasis and antigen-specific CTL activation were not altered between Mn-insufficient and Mn^2+^-administrated *cGas*^*−⁄−*^ or *Tmem173*^*−⁄−*^ mice. These results strongly suggested that Mn is important for preventing tumorigenesis under physiological conditions and that Mn^2+^-mediated antitumor immune responses are dependent on the cGAS-STING pathway. This agreed well with previous findings that Mn is important for cGAS-STING-mediated cytosolic DNA sensing and that cGAS-STING-mediated tumor DNA sensing by tumor-infiltrating DCs is critically required for antitumor immune responses.^13,14,16–20^ Given the facts that Mn^2+^ activates cGAS with 10^4^ times less dsDNA than Mg^2+^ does, Mn^2+^-cGAMP acquires 3.5 × 10^2^ times higher-binding affinity to STING than Mg^2+^-cGAMP, and that Mn-insufficient mice are highly susceptible to DNA viruses,^26^ it is very likely that cytosolic Mn^2+^ is also critically involved in sensitizing cGAS-STING for tumor surveillance under physiological conditions.

We also demonstrated that Mn^2+^ can be used as a potent antitumor reagent or adjuvant. Mn^2+^ administration intranasally, intravenously, or intratumorally induced robust systemic antitumor responses in all tested mouse models, with promoted NK cell function, DC and macrophage maturation/activation, CD8^+^ T cell differentiation/activation and memory T cell survival in tumors, and greatly inhibited tumor growth and metastasis. Moreover, Mn^2+^ synergistically boosted the effects of various antitumor treatments, causing significantly reduced dosage of anti-PD-1 antibodies. Consistently, Mn^2+^ substantially induced the maturation of DCs in PBMCs from various types of cancer patients. More importantly, the phase 1 clinical trial started from November 2018 in patients with advanced metastatic solid tumors, who failed to standard anticancer treatments or to combined chemotherapy or radiotherapy and anti-PD-1 treatment, exhibited favorable clinical efficacy with 45.5% objective response and 90.9% disease control, after additional Mn^2+^ administration. During the submission of this work, a published paper also reported the antitumor effect of Mn^2+^ in mice,^58^ which is consistent with our observation. Therefore, it is promising for Mn^2+^ to improve the therapeutic antitumor effects in the clinic, especially cancer patients with low immunogenicity (cold-tumor) or those with low anti-PD-1 responses. In fact, the development of compounds modulating STING has been the focus of research for cancer treatment. DMXAA, a mouse STING agonist, has been reported to possess strong antitumor activity in mouse but failed in clinical trials because it is unable to activate human STING.^59–62^ cGAMP has been recently proved to restrict tumor progression.^17^ Nevertheless, intravenous injection with high daily doses of cGAMP leads to only modest efficacy due to the poor transmembrane ability of cGAMP,^21^ which usually needs cell permeabilization for its intracellular delivery. However, Mn^2+^ readily activates cells because Mn^2+^ is actively and efficiently transported into cells, with an intracellular Mn^2+^ concentration approximately ten times higher than the extracellular one, by various transporters including the divalent metal transporter (DMT1), Zn^2+^, and Ca^2+^ transporters.^23,26^ Moreover, more recent studies demonstrated that Mn^2+^ directly activates cGAS independent of DNA and triggers a distinct catalytic synthesis of 2′3′-cGAMP,^63,64^ strengthening the potential applications of Mn^2+^ as a novel STING agonist to boost antitumor and antiviral immune responses.

The “normal” range of mammalian tissue Mn concentrations is between 5 μM and 53 μM^65,66^ with blood Mn concentrations within 0.029 μM and 1.2 μM, and brain containing the highest Mn levels from 20 μM to 53 μM.^67^ Previously we observed that THP1 cells, a cell line derived from peripheral blood, started to gain antiviral activity when the culture medium Mn^2+^ level reached 2 µM. Importantly, relatively higher blood Mn levels (between 0.821 μM and 0.96 μM) in five metastatic patients induced acute CRS, resulting in PR (2 patients) or SD (3 patients). Our results indicated that the blood Mn levels required for promoting antitumor therapies were within the physiological range and could be considered relatively safe. In addition, it is known that Mn turnover in plasma is fairly rapid as the estimated half-life for Mn to leave plasma is 1 min^68^ as Mn is promptly redistributed to liver and conjugated to bile and passed to the intestine for fecal excretion.^69,70^

Cancer immunotherapy has recently been conclusively demonstrated to be effective and important for future tumor treatment. However, only small percent of cancer patients respond to immunotherapy presumably because of inadequate immune activation to recognize tumor-specific antigens and generate tumor-specific CD8^+^ T cells. As a result, different types of combination regimens become mainstream practice to extend the clinical benefits of ICBs.^71^ Combination with chemotherapeutics is common in various types of advanced metastatic solid tumors, though its potentiated activity is relatively limited.^72,73^ Prominent clinical response rate from this first-in human trial preliminarily suggested the augmentation effects of Mn^2+^ to the chemo-immunotherapy regimen, which were characterized by the facts: diseases were durably controlled in 20 patients (90.9%) including 10 patients (45.5%) evaluated as PR response, in which 5 patients ever failed to previous anti-PD-1 plus chemotherapies. Strikingly, of eight patients with platinum and/or anti-PD-1 antibody-resistant metastatic ovarian cancer, six achieved PR and two shrunk SD, accompanied with impelling relief of their frozen pelvis in six patients. The noticeable promoting effects of Mn^2+^ to ICB efficiency was evidenced by the elevated blood Mn^2+^ concentrations, which were nicely linked to the induction of type I IFNs, and subsequent release of certain pro-inflammatory cytokines in patients. Among those with markedly increased cytokines (IFNα, IL-6 and TNFα), five patients developed suspected acute local or systemic CRS, 2–3 weeks after their blood Mn^2+^ concentration increased significantly. In fact, CRS has been rarely reported in patients treated only by chemotherapy plus anti-PD-1 antibody.^74–76^ All these clinical findings collectively supported the notion that Mn^2+^ addition effectively boosted adaptive immune responses in patients by activating innate immunity. Based on these results, we proposed that the antitumor immunotherapy would act like driving a vehicle, whereas the ICB functions to release the brake, Mn^2+^ administration presses the accelerator or even starts the engine. Both are essential for an effective antitumor treatment.

Given that Mn^2+^ is an essential nutrient with well-studied toxicology and FDA approved usage by oral, intramuscular and intravenous administrations, we believe that Mn^2+^ has clinical potentials for the development of universal antitumor therapies. Moreover, the component simplicity and steadiness of Mn^2+^, the low cost and wide availability of Mn^2+^ make such therapies even more promising and attractive.

## Materials and methods

### Antibodies for western blot analysis

Anti-GAPDH (Santa Cruz, sc-25778), anti-cGAS (Santa Cruz, sc-245858; Sigma, HPA031700) antibodies were purchased as indicated. Other antibodies are homemade and used as previously described.^77–79^

### Antibodies for flow cytometry analysis

The following antibodies were used (all antibodies from BioLegend unless otherwise indicated): FITC-anti-mouse CD4 (GK1.5), PE/Cy7-anti-mouse CD4 (GK1.5), PE-anti-mouse CD8α (53–6.7), PE/Cy7-anti-mouse CD8α (53–6.7), PerCP/Cy5.5-anti -mouse CD8α (53–6.7), APC-anti-mouse CD8α (53–6.7), FITC-anti-mouse CD3 (17A2), PE/Cy7-anti-mouse CD3 (17A2), Alexa Fluor 700-anti-mouse CD45 (30-F11), APC-anti-mouse CD45 (30-F11), BV510-anti-mouse CD45 (30-F11), PerCP/Cy5.5-anti-mouse CD44 (IM7), PE/Cy7-anti-mouse CD25 (PC61.5, eBioscience), PE-anti-mouse IFN-γ (XMG1.2), BV421-anti-mouse IFN-γ (XMG1.2), FITC-anti-mouse TNFα (MP6-XT22), PE-anti-SIINFEKL-Tetramer (TS-5001-1C, MBL), FITC-anti-mouse CD80 (16-10A1), APC-anti-mouse CD80 (16-10A1), PerpCP/Cy5.5-anti-mouse CD86 (GL-1), APC-anti-mouse CD86 (GL-1), APC/Cy7-anti-mouse I-A/I-E (M5/114.15.2), Alexa Fluor 488-anti-mouse I-A/I-E (M5/114.15.2), PE-anti-mouse CD11c (N418), APC-anti-mouse CD11c (N418), PE-anti-mouse CD45.1 (FC), Alexa Fluor 700-anti-human CD45 (2D1), PE-anti-human CD11c (3.9), APC/Cy7-anti-human HLA-DR (L243), APC-anti-human CD86 (IT2.2), PE-anti-mouse-CD62L (MEL-14), PE-anti-human/mouse Granzyme B (QA18A28), PE /Dazzle594-anti-mouse CD69 (H1.2F3), APC-anti-mouse Perforin (S16009B), Alexa Fluor 488-anti-mouse NK-1.1 (PK136), PE-anti-mouse CD83 (Michel-19), APC-anti-mouse CD107a (1D4B), PE/Cy7-anti-mouse CD86 (P03), BV421-anti-mouse F4/80 (BM8). Flow cytometry data were acquired on an LSR Fortessa flow cytometer (BD Biosciences) and analyzed using FlowJo software (Tree Star).

### Reagents

All chemicals were purchased from Sigma-Aldrich (St. Louis, MO), unless otherwise stated. CFSE cell division tracer kit (Biolegend, 423801), Perm/Wash buffer (BD Biosciences, 51-2091KZ), Red Blood Cell Lysis Buffer (Solarbio, R1010), 4% fixative solution (Solarbio, P1110), LPS (Sigma, L4130), Ovalbumin (InvivoGen, #vac-pova), MHC-I specific OVA peptide H-2K^b^: SIINFEKL (OVA_257–264_) (SciLight Peptide), Collagenase D (Roche, 11088866001), DNase I (Roche, 10104159001), cyclophosphamide monohydrate (TargetMol, T0707), Recombinant Mouse IL-2 (Biolegen, 575404), Recombinant Mouse IL-15 (Biolegend, 566302), Mouse NK Cell Isolation Kit (Miltenyi-Biotec, 130-115-818).

### Cells

B16F10, B16F10-OVA, B16F0-OVA, MC38, LLC, L929 cells (mouse fibroblast cells), L929-ISRE cells (L929 cells expressing an Interferon-Stimulated Responsive Element) were cultured in DMEM; E.G7 cells were cultured in RPMI-1640 medium supplemented with 10% (v/v) fetal bovine serum (FBS), 5 μg/mL of penicillin and 10 μg/mL of streptomycin. To generate BMDCs, bone marrow cells from tibia and femur were flushed out by PBS and plated out in dish, receiving 20 ng/mL of GM-CSF, 20 ng/mL of IL-4 and were incubated at 37 °C in humidified 5% CO_2_ (day 0). On day 3, the plates were replaced by half of the medium containing 20 ng/mL of GM-CSF, 20 ng/mL of IL-4, on day 6, BMDCs were harvested for experiments. Bone marrow derived macrophages (BMDMs) were generated as described.^80^ Briefly, bone marrow cells from tibia and femur were flushed out by PBS cultured in 10 mL medium (DMEM supplemented with 20% heat-inactivated FBS, glutamine, and 30% L929 supernatant containing macrophage-stimulating factor) at 37 °C in humidified 5% CO_2_ for 5 days. Alveolar macrophages were collected from mouse bronchoalveolar lavage fluid. NK cells were enriched from C57BL/6 mouse spleens with mouse NK cell isolation kit per the manufacturer’s instructions. NK cells were cultured for 3 days in RPMI-1640 medium supplemented with 10% FBS in the presence of 20 ng/mL mouse recombinant IL-2 and 50 ng/mL IL-15.

### Human subjects

The study was approved by the Ethical Committee on Human Research of Peking University and the Institutional Review Board of the Chinese PLA General Hospital, and was in accordance with the Declaration of Helsinki. Fifty-one cancer patient volunteers donating blood for characterizing the response of human PBMCs to Mn^2+^ were between 27 and 75 years of age at the time of donating as indicated in the Supplementary information, Table S2. 20 of the volunteers were male and 31 were female. PBMCs were isolated from peripheral blood of volunteers using Histopaque-1077 (Sigma, 10771) through consecutive centrifugation, and cultured in RPMI-1640 media supplemented with 5% FBS. PBMCs were seeded in 24-well plates at a final concentration of 1 × 10^6^ cells/ml.

### Mice

WT C57BL/6 mice were purchased from Beijing Vital River Laboratory Animal Technology Co., Ltd. *cGas*^*−⁄−*^ and *Tmem173*^*−⁄−*^ mice were obtained by crossbreeding the founder mice, which is generated by cytoplasmic injection of C57BL/6 zygotes with Cas9 mRNA (100 ng/μL) and gRNA (50 ng/μL). Cas9 mRNA and single-guide RNA (gRNA) were in vitro-transcribed by mMESSAGE mMACHINE T7 Ultra (Ambion, am1345) and HiScribeTM T7 High Yield RNA Synthesis Kit (NEB, E2040S) respectively. *Rag1*^*−⁄−*^ mice were from Hai Qi (School of Medicine, Tsinghua University), *β2m*^*−⁄−*^ mice were from Zhongjun Dong (School of Medicine, Tsinghua University). OT-I mice were from Yan Shi (School of Medicine, Tsinghua University). All mice were bred and kept under specific pathogen-free conditions in the Laboratory Animal Center of Peking University. Experiments were undertaken in accordance with the National Institute of Health Guide for Care and Use of Laboratory Animals, with the approval of Peking University Laboratory Animal Center, Beijing. Mice were used between 6 weeks and 8 weeks of age.

### Transplant tumor models and treatment

WT C57BL/6 mice were injected subcutaneously into the right groin with 5 × 10^5^ B16F10, 1 × 10^6^ MC38 or LLC or E.G7 cells in 100 μL PBS unless otherwise stated. For the melanoma metastasis model, 2 × 10^5^ B16F10 cells in 300 μL PBS were injected intravenously into C57BL/6. Tumor growth was monitored daily and measured every 1–2 d. Tumor volume was determined as length (mm) × width (mm^2^) × 0.5. For Mn^2+^ treatment, WT-tumor bearing mice were treated intranasally with saline or 5 mg/kg MnCl_2_ once every 2 days. For immune checkpoint blockade therapy, WT tumor bearing mice were treated intraperitoneally with 200 μg α-PD-1 monoclonal antibody (Clone 29 F.1A12, BioXCell) in 200 μL saline on 3, 7, 11 days post tumor inoculation. Control mice received rat IgG2a isotype (Clone 2A3, BioXCell).

### Tumor dissociation

Tumors were minced and digested with 1 mg/mL collagenase D (Roche) supplemented with 10 U/ml DNase I (Roche) for 45 min at 37 °C prior to filtering through a 70-μm cell strainer to obtain single-cell suspensions.

### Tetramer staining

TILs isolated from tumors or splenocytes were stained with SIINFEKL tetramer (MBL) at 4 °C for 1 h, shield from light. Cells were washed twice with staining buffer and followed by staining with anti-mouse CD8α and other surface markers (Biolegend) at 4 °C for 30 min. Cells were washed twice and resuspended in staining buffer for FACS analysis.

### Immunofluorescence

Paraffin slides were de-waxed, rehydrated, subjected to heat-induced epitope retrieval (HIER), and followed by incubation with primary antibody to mouse CD8 (GB11068, Servicebio) or mouse CD4 (GB13064-2, Servicebio). Goat anti-rabbit Alexa Fluor 488 antibody (GB25303, Servicebio) was used as secondary antibody. DAPI (G1012, Servicebio) was used as the nuclear counterstain.

### Bioluminescence imaging

Mice were injected intraperitoneally with D-luciferin (150 mg/kg) and anesthetized 5 mins before the peak of luciferin uptake, then mice were subjected to bioluminescence imaging (BLI) using IVIS Lumina III (PerkinElmer). Luciferase expression was imaged and calculated by Living Image software.

### Viral stock and virus infection

Sendai virus (SeV, from Congyi Zheng, Wuhan University), Vaccinia virus (VACV, Western Reserve strain, from Min Fang, Institute of Microbiology, CAS; or Western Reserve-Vvt7 strain, from Meilin Jin, Huazhong Agricultural University). Virus titer was measured by plaque assay using BHK21 cells. For cell stimulation, cells were infected with SeV (MOI of 0.01), VACV (Western Reserve-Vvt7 strain, MOI of 0.01) for 1 h, rinsed and cultured in fresh medium.

### Type I IFN bioassay

Type I IFN concentration was measured as previously described.^81^ Briefly, an IFN-sensitive luciferase vector was constructed by cloning IFN-stimulated response element (ISRE) into pGL3-Basic Vector (Promega), and stably transfected into L929 cells. L929-ISRE cells were seeded to 96-well plates and incubated with mouse cell culture supernatants. Recombinant mouse IFNβ (R&D Systems) was used as standards. 4 h later, cells were lysed and measured by Luciferase Reporter Assay System (Promega).

### In vitro cytolytic assay

CD8^+^ T cells isolated from spleens of 6–8 weeks old OT-I mice were purified by MojoSort^™^ Mouse CD8 T Cell Isolation Kit (Biolegend, 480008). CD8^+^ T cells were mixed with BMDC and incubated with pre-plated B16F10-OVA-GFP cells (CD8^+^ T:BMDC:Tumor = 2:1:2) with or without the indicated concentrations of MnCl_2_ for 24 h. B6F10-OVA-GFP cells were analyzed by flow cytometry.

### In vivo cytotoxic T-lymphocyte assay

WT C57BL/6 mice were intramuscularly administrated control, OVA (100 μg) alone or with 20 μg MnCl_2_ suspended in PBS with a final injection volume 100 μL at day 0, 7, and 14. At day 21 postimmunization, naïve C57BL/6 splenocytes were labeled with different concentrations of CFSE (5 μM or 0.5 μM) for 10 mins at 37 °C. The stained cells at high concentrations were pulsed with OVA_257–264_ (10 μg/mL) for 90 mins at 37 °C. After washing twice with medium, labeled cells were mixed and transferred to immunized mice by intravenous administration. 24 h after transfer, splenocytes were collected and the percentage of CFSE-labeled cells was measured by flow cytometry. The specific killing percentage was calculated as: (CFSE^low^% – CFSE^high^%)/CFSE^low^% × 100.

### RT-PCR and quantitative PCR analysis

Total RNA was isolated using TRIzol reagent (Invitrogen), according to the manufacturer’s instruction. One microgram of total RNA was converted into cDNA with random primer and Superscript III reverse transcriptase (Invitrogen). PCR was performed with gene-specific primer sets. Quantitative real-time PCR was performed with Sybr green incorporation on the LightCycler^®^ 96 System (Roche), and the data were presented as accumulation index (2^△△^Ct).

### T cell proliferation assay

CD45.1^+^CD8^+^ T cells were purified from OT-I transgenic mice spleens using MojoSort^™^ Mouse CD8 T Cell Isolation Kit. Then CD8^+^ T cells were labeled with CFSE and then washed twice with PBS. On day 0, 2 × 10^6^ CFSE labeled CD8^+^ T cells were transferred intravenously to CD45.2 background recipient mice. On day 3, CD45.1^+^CD8^+^ T cells from spleens of recipient mice were analyzed by flow cytometry to quantify the ratio of CFSE^low^ cells.

### Mouse immunization

Mice were vaccinated with a prime-boost regimen: animals were primed intramuscularly (i.m.) on day 0 with antigen OVA (100 μg) alone or with 20 μg/50 μg MnCl_2_ suspended in PBS with a final injection volume 100 μL. Immunization was boosted on day 7 and day 14.

### Diet-induced Mn insufficiency

Weaning C57BL/6 mice were randomly divided into two dietary groups and fed with either the control diet AIN-76A (D10001, with 59.34 ppm Mn) or AIN-76A without added Mn (D18901, with Mn lower than 1 ppm), obtained from Research Diets Inc. (New Brunswick, U.S.A.) for 6–8 weeks. Feed consumption and body weight of the mice were measured every week. Mice were sacrificed by carbon dioxide asphyxiation followed with cervical dislocation. Mice fed with control diet were indicated as +Mn, Mn-sufficient or control mice, while mice fed with Mn-deficient diet were indicated as –Mn or Mn-insufficient mice.

### The clinical trial

The open-label, dose-escalation and dose-expansion phase 1 clinical trial was approved by the institutional review board of the Chinese PLA General Hospital (S2018-182-01) and was conducted in accordance with the ethical guidelines of the Declaration of Helsinki and the International Conference on Harmonization guidelines for Good Clinical Practice. All the authors vouch that the study protocol was strictly followed and for the accuracy and completeness of the data. Written informed consent was obtained from each enrolled patient.

Eligible patients were adults (aged 18 years or older) with histologically proven advanced metastatic solid tumors; have at least one measurable lesion ≥1 cm as defined by response criteria; an Eastern Cooperative Oncology Group (ECOG) performance status 0–2; adequate organ function; no previous organ transplantation; and no uncontrolled active infection. Patients with a history of relapse following anti-PD-1 or CAR-T therapy were eligible. A complete list of the inclusion and exclusion criteria is provided in the Supplementary information, Table S5.

All patients were administered with Mn chloride intranasally 0.05 or 0.1 mg/kg/d or by inhalation 0.1, 0.2, or 0.4 mg/kg/d once daily, followed by intravenous chemotherapy (day 2) plus 2–4 mg/kg anti-PD-1 antibody (day 3) in a 3-week cycle. Tumor assessments were performed at the baseline and every 6 weeks by computed tomography (CT) and/or magnetic resonance imaging (MRI). Tumor responses were assessed in a blinded manner according to the Response Evaluation Criteria in Solid Tumors (RECIST) v1.1. MRI of the brain and neurological examination was performed to assess the safety of Mn^2+^ treatment. All adverse events were monitored and documented according to the Common Terminology Criteria for Adverse Events, version 5.0.

### Free blood Mn^2+^ analysis

The blood samples were collected before each cycle in heparinized tube. 20 μL sample centrifuged at 4000 rpm for 1 min after standing 15 min for the trace element activation. The concentration of free blood Mn^2+^ was detected with High Precision Trace Element Analyzer (AASA medical technology Co., Wuhan, China), which was approved by the National Medical Products Administration of China (2013-2401836). The normal range of free blood Mn^2+^ was 0.029–1.2 μM.

The determination of blood Mn^2+^ concentration was based on an electrochemical technology Differential-pulse Polarographic Determination. This is a trace element detection technology with different working principles from inductively coupled plasmaatomic emission spectroscopy (ICP-AES) recommend by ATSDR. The blood samples were pretreated with sulfosalicylic acid to destroy and precipitate the protein and other macromolecular, which would contribute to the release of Mn from bond state to free state. According to NMPA, the normal ranges of blood Mn^2+^ are 0.029–1.2 μM by polarographic determination and 0.06–0.38 μM by ICP-AES in China, respectively.

### Serum cytokine and chemokine analysis

All blood samples were collected in nonheparinized tubes and allowed to clot at room temperature for 2 h and then centrifuged at 1500× g for 10 min. Serum samples were absorbed from the coagulated blood and stored at −80 °C. Serum cytokines and chemokines were analyzed by LEGENDplex bead-based immunoassays (BioLegend, San Diego, USA) according to the manufacturer’s instructions. The human CD8/NK panel (740267, Biolegend) were used to simultaneously quantify 13 serum cytokines/chemokines, including IL-2, 4, 6, 10, 17A, IFNγ, TNFα, soluble Fas (sFas), soluble FasL (sFasL), granzyme A, granzyme B, perforin and granulysin. The human antiviral response panel (740349, Biolegend) was used to analyze 11 human serum inflammatory cytokines/chemokines, including IFN-α, β, γ, IL-1β, 6, 8, 10, 12, TNFα, IP-10 and GM-CSF. Data acquisition was performed on a BD FACSCalibur flow cytometer (BD Biosciences) and analyzed with the LEGENDplex^™^ Data Analysis Software (BioLegend).

### Statistical analysis

The Student’s *t*-test was used to analyze data. The compared two groups were labeled by square brackets. Survival curves were compared using the Mantel-Cox test. For the bar graph, one representative experiment of at least three independent experiments is shown, and each was done in triplicate. Descriptive statistics and Wilson method was used to summarize the proportion of patients with response and calculated the 95% CIs, respectively. Differences between treatment cycles were detected by paired *t*-tests. All statistical analyses were performed in SPSS 25 unless otherwise stated. For the dot plot graph, each dot point represents one independent biological replicate. Data are shown as means ± SEM (*n* ≥ 3). ns, not significant, *P* > 0.05; *P < 0.05; **P < 0.01; ***P < 0.001; ****P < 0.0001.

## Supplementary information

Supplementary information, Data S1

Supplementary information, Fig. S1

Supplementary information, Fig. S2

Supplementary information, Fig. S3

Supplementary information, Fig. S4

Supplementary information, Fig. S5

Supplementary information, Fig. S6

Supplementary information, Fig. S7

Supplementary information, Fig. S8

Supplementary information, Table S1

Supplementary information, Table S2

Supplementary information, Table S3

Supplementary information, Table S4

Supplementary information, Table S5

Supplementary information, Table S6

## Data Availability

All data supporting the findings of this study are available within the paper and its supplementary materials. RNA-seq data have been deposited in Gene Expression Omnibus under accession no. GSE126670.
