# Supplementary material for: Manganese is critical for antitumor immune responses via cGAS-STING and improves the efficacy of clinical immunotherapy
Source: Cell Res. 2020 Aug 24;30(11):966–79. doi: 10.1038/s41422-020-00395-4 (PMC7785004; doi:10.1038/s41422-020-00395-4)
Supplement: Supplementary file 1 — Supplementary information, Data S1 [file 41422_2020_395_MOESM1_ESM.pdf]

Protocol

A First-in-human Safety Study of the Pan-immunotherapy in Patients with Unresectable/Metastatic Solid Tumors or Lymphomas

Table of contents

Title Page 4
Summary 6
1. Introduction 9
    1.1 Background and Rational 9
2. STUDY OBJECTIVES 11
    2.1 Research hypothesis 11
    2.2 Objectives 11
3. ETHICAL CONSIDERATIONS 11
    3.1 Ethical conduct of the study 11
    3.2 Subjects data protection 12
    3.3 Informed Consent 12
    3.4 Change to the protocol and informed consent form 13
    3.5 Audits and inspections 14
4. Selection of subjects 14
    4.1 Inclusion criteria 14
    4.2 Exclusion criteria 16
    4.3 Criteria for termination of treatment 16
    4.4 Criteria for removal from study 17
5. Study design 17
    5.1 Overall design 17
    5.2 Baseline evaluation 18
    5.3 Studies during treatment 19
    5.4 Treatment duration 19
    5.5 Studies on completion of therapy 20
    5.6 Follow-up 20
    5.7 Response criteria 20
6. Treatment plan 21
    6.1 Manganese administration 21
    6.2 Anti-PD-1 antibody plus chemotherapy treatment 21
    6.3 Dose modification 22
    6.4 Maintenance therapy 22
    6.5 Concomitant treatments 22
7. investigational products 23
    7.1 General information 23
    7.2 Drug label 23
    7.3 Handling and Dispensing 23
    7.4 Preparation and administration 24
8. Safety reporting requirements 24

8.1 Definition of adverse events (AE) ... 24
8.2 Treatment-related AE ... 25
8.3 Immune-mediated AE ... 25
8.4 Manganese-related AE ... 25
8.5 Serious AE (SAE) ... 26
8.5 Grading and recording of AEs ... 26
8.6 The report of deaths ... 27
8.7 Management of the treatment related toxicities ... 28
9. Statistical analyses ... 28
9.1 Statistical considerations ... 28
9.2 Sample size determination ... 29
9.3 Outcome assessment ... 29
9.4 Safety assessment ... 30
9.5 Blood Manganese concentration assessment ... 30
9.6 Peripheral blood immune cell and serum cytokine/chemokine assessment ... 30
9.7 Data analysis ... 31
List of abbreviations ... 32
Reference ... 34
Appendix 1. Time and event schema ... 38
Appendix 2. Tumor response criteria ... 39
1. Time point response: patients with target (+/–non-target) disease ... 39
2. Best overall response when confirmation of CR and PR required ... 39
3. Measured dominant lesions ... 40
4. Non-measured lesions ... 40
Appendix 3. Common terminology criteria for adverse events (CTCAE), v5.0 ... 41
Appendix 4. ECOG performance status scale ... 42
Appendix 5. New York heart association (NYHA) classification ... 42

Title Page

Camrelizumab plus GVD Chemotherapy in Rituximab-refractory Primary Mediastinal B-cell Lymphoma

Principal Investigator Weidong Han M.D., Ph.D.
Department of Bio-therapeutic
Department of Molecular Biology and Immunology
Chinese PLA General Hospital
Beijing 100853 China

Co-Investigator Qian Mei Ph.D.
Meixia Chen M.D.
Yan Zhang M.D.
Kaichao Feng M.D.
Yang Liu M.D.
Qingming Yang M.D.
Jiejie Liu B.D.
Jingying Zhang B.D.
Xiang Li B.D.
Liang Dong B.D.

Regulatory Sponsor Weidong Han M.D., Ph.D.
Department of Bio-therapeutic
Department of Molecular Biology and Immunology
Chinese PLA General Hospital
Beijing 100853 China

Funding Sponsor National Key Research and Development Program of China
National Natural Science Foundation of China
Fostering Funds of Chinese PLA General Hospital for National Excellent Young Scholar Science Fund

Study Product Manganese Chloride; anti-PD-1 antibody; Chemotherapy

Original Protocol Oct 25, 2018

CONFIDENTIAL

This document is confidential and the property of Chinese PLA General Hospital. No part of it may be transmitted, reproduced, published, or used by other persons without prior written authorization from the study sponsor.

STUDY TEAM

Clinical Trial Site - Chinese PLA General Hospital

Principal Investigator

Weidong Han M.D., Ph.D.

**Clinical Team**	**Laboratory Team**
Meixia Chen M.D.	Qian Mei Ph.D.
Yan Zhang M.D.	Jiejie Liu B.D.
Kaichao Feng M.D.	Jingying Zhang B.D.
Yang Liu M.D.	Xiang Li B.D.
Qingming Yang M.D.	Liang Dong B.D.
Fengxia Shi BSN	

Sponsor Cell Manufacturing-Chinese PLA General Hospital

Weidong Han PhD Medical Director & Laboratory Director

Summary

Protocol title: A First-in-human Safety Study of the Pan-immunotherapy in Patients with Unresectable/Metastatic Solid Tumors or Lymphomas

Investigational Product(s): Manganese Chloride Administered intranasally in Arm 1 (0.05, 0.1 or 0.2 mg/kg/d) and by inhalation in Arm 2 (0.1, 0.2 or 0.4mg/kg/d) once daily, combined with chemotherapy (Day 2) plus 2-4 mg/kg anti-PD-1 antibody (Day 3) intravenously in a 3-week cycle

Study objectives:

Primary objectives:

To determine the mode of delivery of manganese, and assess the safety and tolerability of manganese priming anti-PD-1 antibody plus chemotherapy.

Secondary objectives

To evaluate the preliminary efficacy by objective response rate (ORR) and disease control rate (DCR), and the q3w pharmacokinetic profile of manganese.

Exploratory objectives

To detect the phenotype and activity of peripheral immune cells, serum cytokines and chemokines before and during the treatment of combination therapy, and assess the correlation with the outcomes.

Treatment plan

Subjects will receive study drugs as Figure 1.

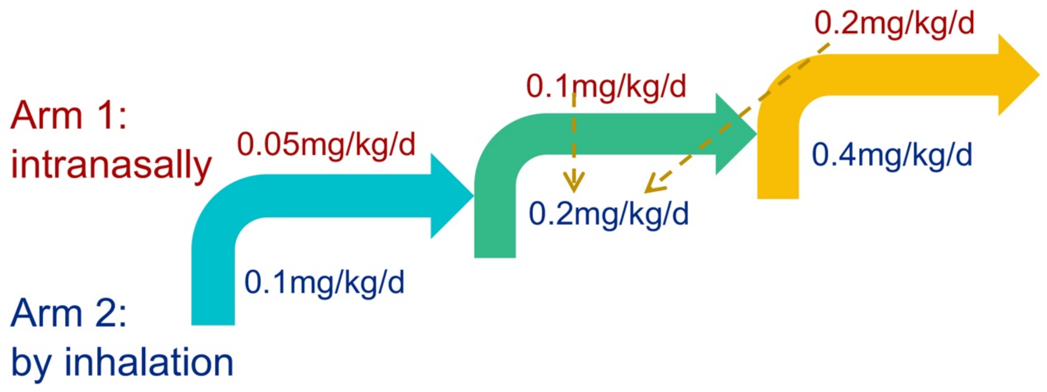

Figure 1. Treatment plan

Study Population

Subjects must meet all eligibility criteria, including the following:

Key inclusion criteria:

1) Subjects with histologically proven unresectable/ metastatic solid tumors or lymphomas who refractory to or relapsed from at least two frontline therapies.

2) The patients initially diagnosed with local advanced or metastatic pancreatic cancer or cholangiocarcinoma could be enrolled without prior treatment.

2) Adults.

3) Subjects must have at least one measurable lesion ≥ 1 cm.

4) ECOG performance of 0 or 2.

5) Subjects must be off other anticancer therapy for at least 4 weeks prior to Day 1.

6) Subjects must have adequate bone marrow, live, renal, lung and heart functions.

7) Signed informed consent.

Key exclusion criteria:

1) T cell lymphomas or leukemia.

2) Subjects with any autoimmune disease or history of syndrome that requires corticosteroids or immunosuppressive medications.

3) Serious uncontrolled medical disorders or active infections, pulmonary and intestinal infection especially.

4) Active alimentary tract hemorrhage or history of alimentary tract hemorrhage in 1 month.

5) Prior organ allograft or allogeneic bone marrow transplantation.

6) Other antitumor antibodies within 4 weeks, previous treatment with anti-PD-L1, anti-PD-L2, anti-CTLA-4 or agents targeting T-cell co-stimulation.

7) Unsuitability for the trial, based on clinical judgment.

Study assessment

Safety evaluation:

Adverse events will be assessed continuously during the study and for 3 months post last treatment. Adverse events will be evaluated according to the NCI CTCAE, Version 5.0. Subjects should be followed until all treatment-related adverse events have recovered to baseline or are deemed irreversible by the investigator.

Efficacy evaluation:

Objective response rate (ORR) and disease control rate (DCR) will be evaluated using the Response Evaluation Criteria for Solid Tumors (RECIST version 1.1).

Experimental biomarkers detection:

Available blood samples will be collected at baseline and specified time points for all subjects. The peripheral immune cell phenotype, activity and serum cytokines/chemokines were detected by FACS.

1. Introduction

1.1 Background and Rational

In the past decade, our understanding of the interactions between tumors and the immune system has been profoundly advanced. Many pre-clinical and clinical success has witnessed the development of therapeutic approaches that boost the body's natural defenses against cancer[1, 2]. These therapies are aimed to mount effective antitumor immune responses and include immunomodulators, vaccines, and adoptive transfer of immune cells[3]. Reactivation of cytotoxic CD8+ T-cell responses has set a new direction for cancer immunotherapy. Some of the most clinically effective immunotherapies to date target the immune checkpoint programmed cell death protein 1 (PD-1)[4]. PD-1 is expressed by T cells during priming or expansion and binds to one of its two ligands PD-L1/L2. The interaction of PD-L1/L2 with PD-1 expressed on tumor-infiltrating T cells lead to T-cell anergy and exhaustion, which consequently caused tumor immune escape[5, 6]. Neutralizing monoclonal antibodies against PD-1 can restore an adequate immunosurveillance against the neoplasm and enhance T-cell-mediated antitumor response[7-9].

The blockade to PD-1 has transformed the therapeutic landscape of a wide range of cancers, being particularly successful for tumors with limited therapeutic options[10-13]. However, a significant fraction of patients manifests innate or acquired resistance to these therapies[4, 10]. The limited objective response rate (about 20%) emphasize the need of developing rational combination strategies to obtain more potent anticancer responses.

In 2013, Daniel Chen and Ira Mellman proposed "cancer-immunity cycle", by which the anti-cancer immune responses lead to an effective elimination of cancer cells. The cycle includes a series of self-sustaining stepwise events: 1) release of tumor antigen, 2) presentation of tumor antigen, 3) T-cell priming and activation, 4) trafficking of T-cells to tumors, 5) T-cell infiltration into tumors, 6) recognition of cancer cells by T-cells, and 7) killing of cancer cells[14]. The cancer-immunity cycle

may help explain and provide guidelines for potential enhancement. In the case of anti-PD-1 therapy, a variety of biological factors contribute to treatment resistance, including lack of cancer antigens recognizable by T cells, impaired cancer-antigen presentation, impaired activation of cancer-specific T cells, and so on[15-17]. Therefore, to achieve improved immune responses, combination strategies of anti-PD-1 antibody with other therapeutics that augment functions at the above-mentioned different stages of cancer immune cycle seems to be a rational resolution.

Expression of tumor-associated antigens on cancer cells is one of the tumorigenesis characteristics[18, 19]. Absence of tumor antigen has been demonstrated as one of the upmost reasons why a tumor would not respond to anticancer immunotherapy. Chemotherapy potentiate tumor immune-related responses by harnessing the released tumor antigens from necrotic tumor cells and emitting damage-associated signal[20-22]. It is verified in various cancers that combinatorial chemoimmunotherapy could induce immunogenic tumor phenotype in tumor cells, and also increase the infiltration of T-cells, thus significantly promoted the antitumor immunity[23-25].

Recent works demonstrated that various antitumor therapies depend on the activation of the cGAS-STING pathway, as tumor-derived genomic DNA was found in the cytosol of the tumor-infiltrating DCs to activate this pathway to promote tumor-specific antigen presentation and CTL activation[26-28]. Our previous work confirmed that manganese functions as the activator of cGAS-STING signal and cancer surveillance[29]. Manganese could promote DC maturation and tumor-specific antigen presentation, augmenting CD8+ T cell differentiation and activation, and enhancing memory CD44hiCD8+ T-cell survival[29]. The pre-clinical trial demonstrated that intranasal manganese administration systemically and synergistically boosted antitumor therapies and significantly reduced the amounts of anti-PD-1 antibodies and chemotherapeutic agents required.

Based on this, the manganese priming anti-PD-1 antibody plus chemotherapy should be a rational treatment choice for patients with unresectable/metastatic solid tumors or lymphomas.

2. STUDY OBJECTIVES

2.1 Research hypothesis

Manganese could play a critical role in bridging innate and adaptive immunity for tumor surveillance, and manganese priming anti-PD-1 antibody plus chemotherapy could serve as efficient chemoimmunotherapy with manageable safety profile for patients with unresectable/metastatic solid tumors or lymphomas.

2.2 Objectives

2.2.1 Primary objectives

To assess the safety and tolerability of manganese priming anti-PD-1 antibody plus chemotherapy.

To determine the mode of delivery of manganese.

2.2.2 Secondary objectives

To evaluate the preliminary efficacy by objective response rate (ORR) and disease control rate (DCR).

To assess the q3w pharmacokinetic profile of manganese.

2.2.3 Exploratory objectives

To detect the phenotype and activity of peripheral immune cells, serum cytokines and chemokines before and during the treatment of combination therapy, and assess the correlation with the outcomes.

3. ETHICAL CONSIDERATIONS

3.1 Ethical conduct of the study

This is a phase I, single-center, two-armed, open-label, multiple dose, first in human study clinical trial. The trial was conducted in accordance with the ethical guidelines of the 1975 Declaration of Helsinki and with Good Clinical Practice guidelines provided by the International Conference on Harmonization. The study

was approved by the Institutional Review Board (IRB) of the Chinese PLA General Hospital (review broad identifier, S2018-182-01).

The IRB will also function as data and safety monitoring committee.

The study will be conducted in compliance with the protocol. The protocol and any amendments and the subject informed consent will receive IRB approval/favorable opinion prior to initiation of the study.

Personnel involved in conducting this study will be qualified by education, training, and experience to perform their respective tasks.

Principal investigator is responsible for providing IRB with reports of any serious and unexpected adverse drug reactions from any other study conducted with the investigational product. The research team will provide this information to the principal investigator so that he can meet these reporting requirements.

3.2 Subjects data protection

The Informed Consent Form will incorporate wording that complies with relevant data protection and privacy legislation.

Precautions are taken to preserve confidentiality and prevent data being linked to the identity of the subjects. In exceptional circumstances, however, certain individual might see both the data and the personal identifiers of a subject. Regulatory authorities may require assess to the relevant files, though the subject's medical information and the genetic files would remain physically separate.

3.3 Informed Consent

Written informed consent to participate was obtained from each patient before enrollment. Investigators must describe the protocol, alternative therapies, and the risks and benefits of each to the individual signing the consent. The investigational nature and research objectives of this trial, the procedures and treatments involved and their attendant risks and discomforts and potential benefits, and alternative therapies will be clearly and fully informed to the patient. The attached informed

consent contains all elements required for consent. In addition, the Principal Investigator or his designee will be available to answer all patient questions throughout their participation in the protocol.

Investigators must: 1) Provide a copy of the consent form and written information about the study in the language in which the subject is most proficient prior to clinical study participation. The language must be non-technical and easily understood. 2) Allow time necessary for subject or subject's legally acceptable representative to inquire about the details of the study. 3) Obtain an informed consent signed and personally dated by the subject or the subject's legally acceptable representative and by the person who conducted the informed consent discussion. 4) Obtain the IRB's written approval/favorable opinion of the written informed consent form and any other information to be provided to the subjects, prior to the beginning of the study, and after any revisions are completed for new information. 5) Revise the informed consent whenever important new information becomes available that is relevant to the subject's consent. The Principal Investigator or his designee should fully inform the subject of all pertinent aspects of the study and of any new information relevant to the subject's willingness to continue participation in the study. This communication should be documented.

The rights, safety, and well-being of the study subjects are the most important considerations and should prevail over interests of science and society.

3.4 Change to the protocol and informed consent form

If there are any substantial changes to the study protocol, then these changes will be documented in study protocol amendment and where required in a new version of the study protocol.

The amendment in to be approved by IRB and if applicable, also the national regulatory authority approval, before implementation. Local requirements are to be followed for revised protocols.

3.5 Audits and inspections

Ethics Committee performs audits or inspections at the center, including source data verification. The purpose of an audit or inspection is to systematically and independently examine all study-related activities and documents, to determine whether these activities were conducted, and data were recorded, analyzed, and accurately reported according to the protocol, Good Clinical Practice, guideline of the International Conference on Harmonization, and any applicable regulatory requirements.

4. Selection of subjects

4.1 Inclusion criteria

Patients must fulfill all of the following inclusion criteria to be eligible for admission to the study:

1) ≥ 18 years old.
2) Patients with histologically proven unresectable/ metastatic solid tumors or lymphomas.
3) Life expectancy of at least 6 months.
4) Patients must have at least one measurable lesion ≥ 1 cm as defined by the Response Evaluation Criteria for Solid Tumors (RECIST version 1.1).
5) Subjects must be refractory to or relapsed from at least two frontline therapies, except for patients initially diagnosed with local advanced or metastatic pancreatic cancer or cholangiocarcinoma.

   Refractory was defined as a lack of response to, or progression during the frontline treatments.

   Relapsed was defined as recurrence within 6 months after the frontline treatments.
6) Eastern Cooperative Oncology Group (ECOG) performance 0 − 2.
7) Subjects must be off other anticancer therapy for at least 4 weeks prior to Day 1.

The patient must have sufficiently recovered from any treatment-related toxicities, except for alopecia.

8) Subjects failure with autologous hematopoietic stem-cell transplantation (ASCT), anti-PD-1 or CAR-T therapy are eligible which must be more than 4 weeks.

9) Subjects must have adequate bone marrow, live, renal, lung and heart functions:

   WBCs ≥ 2000/μL;

   Absolute neutrophil count (ANC) ≥ 1500/μL;

   Platelets ≥ 70 x 10^3/μL;

   Hemoglobin ≥ 7.0 g/dL;

   Serum creatinine of ≤ 1.5 × ULN or creatinine clearance > 40 mL/minute;

   AST ≤ 1.5 × ULN;

   ALT ≤ 1.5 × ULN;

   Total bilirubin ≤ 1.5 × ULN (except subjects with Gilbert Syndrome who must have total bilirubin < 3.0 mg/dL);

   Myocardial enzyme ≤ 1.5 × ULN;

   minimum level of pulmonary reserve defined as ≤ Grade 1 dyspnea and pulse oxygenation > 91 % on room air;

   No clinically significant pleural effusion.

10) No active symptomatic ischemic heart disease, myocardial infarction or congestive heart failure within the past year. Cardiac ejection fraction ≥ 50%, no evidence of pericardial effusion as determined by an ECHO, and no clinically significant ECG findings.

11) Women of childbearing potential must have a negative serum or urine pregnancy test (minimum sensitivity 25 IU/L or equivalent units of HCG) within 3 days prior to the first dose, and must not be lactating.

12) Women and men of childbearing potential must agree to use an adequate method of contraception for the course of the study through 120 days after the last dose of investigational product.

13) Subjects must sign written informed consent in accordance with regulatory guidelines. This must be completed before the administration of protocol related

procedures. The consent includes scheduled visits, treatment schedule and a series of laboratory tests.

4.2 Exclusion criteria

1) Subjects with a history of central nervous system involvement by cancer.
2) T cell lymphomas or leukemia.
3) Treatment with inhibitors of CYP3A4, CYP2C8 or P-gp within 2 weeks prior to Day 1.
4) Subjects with any autoimmune disease or history of syndrome that requires corticosteroids or immunosuppressive medications.
5) Serious uncontrolled medical disorders or active infections, pulmonary and intestinal infection especially.
6) Active alimentary tract hemorrhage or history of alimentary tract hemorrhage within 1 month.
7) Prior organ allograft or allogeneic bone marrow transplantation.
8) Other antitumor antibodies within 4 weeks, previous treatment with anti-PD-L1, anti-PD-L2, anti-CTLA-4 or agents targeting T-cell co-stimulation.
9) Positive for HIV, active hepatitis and syphilis.
10) History of Grade 4 anaphylactic to monoclonal antibody treatment, or known to be allergy to any component of investigational agents previously.
11) Upon the judgment by the investigator, subjects have other factors that possibly cause the halfway-termination of this study, such as other serious illnesses (including mental illness) that require concomitant treatment, serious laboratory abnormalities, with family or social factors, which may influence the safety of the subject, or the collection of trial data and samples.

4.3 Criteria for termination of treatment

1) Subject decision. The subject is at any time free to discontinue treatment, without prejudice to further treatment. Document the reason(s) for stopping

treatment.

2) A serious adverse event that requires the subject's being withdrawn from the trial. In this event, report the severe adverse events and document the reason(s) for withdrawal.
3) Objective disease progression or subjects is no longer receiving clinical benefit.
4) Severe non-compliance with the study protocol as judged by the investigator.
5) Subjects has received the maintenance regimen for 1 year and achieved persistent objective response.

4.4 Criteria for removal from study

Once a subject has received the treatment, subjects should be followed until the subject withdraws consent, dies or are lost to follow up. Below are examples for premature discontinuation include:

1) Rapid progression requiring alternative medical or surgical intervention.
2) Subjects lost to follow-up.
3) At any time, the patient is found to be ineligible for the protocol as designated in the section on Criteria for Patient/Subject Eligibility (i.e., a change in diagnosis), the patient will be removed from the study.

5. Study design

5.1 Overall design

This two-arm, open-label, single-center, phase II trial was designed to determine the mode of delivery of manganese and assess the safety and efficacy of the combined regimen of manganese primed anti-PD-1 antibody plus chemotherapy. Manganese will be provided as Manganese Chloride. Patients with advanced or metastatic solid tumors of any origin and lymphoma can be enrolled. The primary objectives are the toxicity and tolerability of the regimen. The secondary objectives include various efficacy parameters, such as objective response rate (ORR) and disease control rate

(DCR), and the q3w pharmacokinetic profile of manganese.

This study includes the screening phase, treatment phase and follow-up phase. Baseline evaluation was performed in the screening phase, and the subjects met the eligibility criteria would process to the subsequent treatment phase.

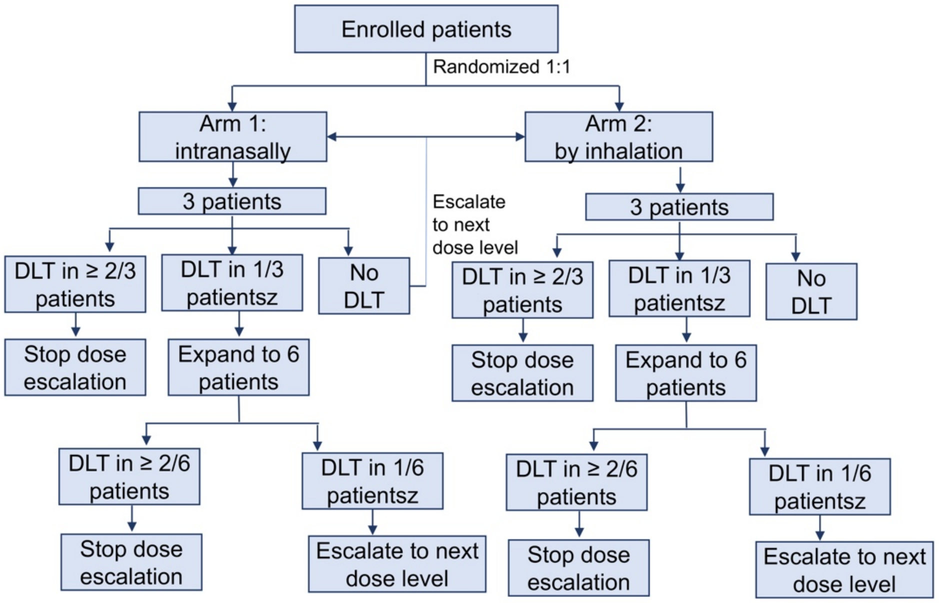

Figure 2. Trial design.

5.2 Baseline evaluation

Once a subject met the initial eligibility criteria via outpatient service, they will be asked to sign of the informed consent form and obtain a subject ID. The baseline evaluation must be performed.

1) Baseline disease, treatment history and tumor assessment including formal documentation of measurable lesions should be performed within 3 weeks.
2) Hb, WBC, differential, platelets, prothrombin time, partial thromboplastin time.
3) Blood manganese concentration.
4) LDH, alkaline phosphatase, bilirubin, albumin, calcium, phosphate, uric acid, BUN, creatinine, and electrolytes.
5) ECHO to assess cardiac ejection fractions as clinically indicated.
6) Electrocardiogram.
7) Pregnancy test in women of childbearing age.

8) Infection disease scteening: HIV: serology; HBV: HBsAg, Anti-HBs, HBeAg, Anti-HBc, Anti-HBe, HBV DNA ≥ 10^4/ml, hepatocyte transaminase; HCV: HCV antibodies and HCV RNA.
9) Computed Tomography (CT) and/or Magnetic Resonance Imaging (MRI) of the target lesions.

5.3 Studies during treatment

1) The clinical response will be evaluated by CT and/or MRI every 2 cycles per the Response Evaluation Criteria in Solid Tumors (RECIST), version 1.1.
2) Adverse event assessments should be documented before each cycle of treatment.
3) All of the clinical exam and biochemistry tests will be collected on Days 1 (before day-1 administration) of each cycle.
4) Peripheral blood samples (10 ml) will be collected on day 1 of each cycle (before day-1 administration) for the phenotype and activity analysis of peripheral immune cells via flow cytometry.
5) Five milliliter of serum samples will be collected on day 1 (before day-1 administration) of each cycle for the serum cytokines and chemokines analysis.

5.4 Treatment duration

1) Study drug is administered up to 8 cycles.
2) Patients achieving CR will receive 4 more cycles for a second radiographic confirmation.
3) The subjects, who achieve confirmed CR or obtain PR after 8 cycles of combination treatment, will receive the maintenance manganese primed anti-PD-1 antibody.
4) Early termination of the treatment of the combined regimen will allowed when disease progression, serious toxicity, withdrawal of consent.

5.5 Studies on completion of therapy

On completion of therapy, all initially positive or abnormal the clinical exam, biochemistry test, radiologic imaging studies will be repeated. CT and/or MRI should be performed. Patients who are taken off protocol will continue to be followed for long-term safety and survival.

5.6 Follow-up

1) The follow-up of safety will be performed within the first 3 months from the last cycle of treatment. Beyond 3 months, patients could be followed for ongoing drug-related events until start of another anti-tumor treatment.
2) The subjects who discontinue the study for non-progression of disease will be included in the follow-up of response duration until disease progression, lost to follow-up, withdrawal of study consent, or start of a subsequent anti-cancer therapy. Radiographic assessments will be performed every 3 months during the first follow-up year, every 6 months during the second year, every 12 months during the third year, and yearly thereafter.
3) All patients will be followed for survival every 3 months until death or withdrawal of study consent.

5.7 Response criteria

From the investigators review of the imaging scans, the tumor response data will be used according to the Response Evaluation Criteria in Solid Tumors (RECIST), version 1.1. The Criteria could be found in Appendix. It will also be used to determine the endpoints ORR and DCR from the overall visit response and scan data.

At each visit, subjects will be assigned a RECIST visit response of CR, PR, SD or PD depending on the status of their disease compared with baseline and previous assessment. If a subject has a tumor assessment that cannot be evaluated then the subject will be assigned a visit response of not evaluable (NE) (unless there is evidence of progression in which case the response will be assigned as PD).

Additional imaging assessments may be performed at any time during the study when the investigator decided to support the efficacy evaluations for a subject, as necessary.

6. Treatment plan

6.1 Manganese administration

The enrolled subjects will be randomized in a 1:1 ratio to one of the following subgroups:

Arm 1: Manganese Chloride administered intranasally daily at 3 dose levels (0.05, 0.1 or 0.2 mg/kg/d);

Arm 2: Manganese Chloride administered by inhalation daily at 2 dose levels (0.2 or 0.4 mg/kg/d).

At least 3 patients will be enrolled at each dose level of each arm. Doses will be increased as the above indicated levels. There will be no further dose escalation once 2 or more patients out of 3 to 6 patients at a certain dose level experience a dose-limiting toxicity (DLT) during the first treatment cycle (one cycle is 21 days). The highest dose level at which not more than 1 out of 6 patients experience a DLT will be the Maximum Tolerated Dose (MTD). The Phase 2 Dose (RP2D) will be determined based on all available safety and blood Mn^{2+} concentration data. If considered needed, each dose level can be extended with additional patients. Based on efficacy and safety data, it may be decided to transfer patients from Arm 1 to Arm 2. The dose for transferred patients will be started at the initial level of the inhalation group (0.2 mg/kg/d).

6.2 Anti-PD-1 antibody plus chemotherapy treatment

Enrolled patients will receive intravenous chemotherapy (day 2) plus anit-PD-1 antibody (2-4mg/kg, day 3) in a 3-week cycle. The chemotherapy will be chosen according to the treatment regimen before enrollment.

6.3 Dose modification

1) Dose modification of anti-PD-1 antibody is not allowed.

2) Modification of chemotherapy doses was done according to the locally approved product information.

- ANC ≥ 1000/μL and platelets ≥ 50 x 10^3/μL on day 21, begin treatment on time;
- ANC < 1000/μL and/or platelets < 50 x 10^3/μL on day 21, hold the next dose for 1 week;
- If counts still low after 1-week delay, decrease 20% dose level of last cycle.

6.4 Maintenance therapy

The subjects achieving the second confirmed CR or obtaining PR after 8 doses would be followed by the maintenance of manganese primed anti-PD-1 antibody treatment.

If a patient is unable to complete all 8 cycles of the combined treatent, and the principal investigator determines that it is in the patient's best interest; the patient would advance into the maintenance therapy.

6.5 Concomitant treatments

1) The short or long acting granulocyte colony stimulating factor (G-CSF) can be given if the patients suffer severe neutropenia (above grade 3) and stopped 24 hours before treatment.
2) Hydration and alkalization as routine treatment during chemotherapy will be allowed.
3) Subjects are permitted the use of topical corticosteroids except intravenous medication. When adverse events happened, systemic corticosteroids are

permitted in a short period of time.

4) Appropriate auxiliary drug, such as liver-protective, heart-protective and stomach-protective, are permitted to perform concomitant with investigational drugs.

5) Hormonal therapy, immunotherapy regimens or concurrent immunosuppressive agents should NOT be used as pre-medication, during treatments and following stage.

6) Anti-tumor drugs and adjuvant drugs related to tumor therapy should be discontinued during treatments, including anti-tumor traditional Chinese medicine and immunological preparations.

7. investigational products

7.1 General information

In this protocol, investigational products are manganese, anti-PD-1 antibody and chemotherapy. Non-investigational products used in this study was support or escape medication for preventative, diagnostic, or therapeutic reasons.

7.2 Drug label

The study drugs will be packaged according to current good manufacturing practice requirements. Labels will be prepared in accordance with Good Manufacturing Practice and local regulatory guidelines. Label text will be provided in Chinese.

7.3 Handling and Dispensing

Management and distribution of the investigational product for this study will be performed by designated person. All the investigational products are only used for the study subjects who are included in this clinical trial. The dosage and administration method should be in accordance with the protocol. The distribution and recovery of each drug shall be recorded on a dedicated record sheet in a timely manner.

The investigational products should be stored in a secure area according to storage requirements. If concerns regarding the quality or appearance of the study drug arise, do not dispense the study drug and contact the sponsor immediately.

7.4 Preparation and administration

1) Manganese Chloride is provided as 1M sterile, nonpyrogenic solution. Dilute to the appropriate concentration with distilled water before each use. For patients in Arm 1, the Manganese Chloride is administrated intranasally using the upper body or lateral head position. The nose drops will last 20-30 min for each time. For patients in Arm 2, the Manganese Chloride is given by inhalation, and lasting 10-20 min each time.
2) The chemotherapeutic drugs, gemcitabine, vinorelbine and doxorubicin, have been marketed. For the detailed preparation method, see the commercial package insert.
3) Anti-PD-1 antibody is stored at refrigerated temperature (2-8°C). Each vial of sterile lyophilized powder shall be reconstructed in distilled water for injection. The distilled water shall be added slowly along the wall of the vial. Visually observe and ensure that the solution is clear, colorless and free from particulate matter. Any partial vials should be safely discarded per the sites standard operating procedures (SOPs) and should not be reused. Intravenous infusion shall be performed with a medical infusion pump using an infusion set with an online filter (0.2 μM) within 2 hours after dilution.

8. Safety reporting requirements

8.1 Definition of adverse events (AE)

An adverse event is defined as any harmful event, reaction, side effect, or untoward occurring in a patient or clinical trial subject, a whether or not considered causally related to the investigational products. A new illness, symptom, sign or

clinically significant laboratory abnormality or worsening of a pre-existing condition or abnormality is considered an AE. An AE not only includes an undesirable medical condition, but also involves run-in or washout periods, even if no study treatment has been administered in clinical studies. All the AE encountered during the study, observed by the doctor or reported by the patient, will be recorded on the case report form in the section provided for this purpose.

8.2 Treatment-related AE

Treatment-related AE means any adverse event for which there is a reasonable possibility that the drug caused the adverse event, which means there is evidence to suggest a causal relationship between the drug and the adverse event. This implies a lesser degree of certainty about causality than adverse reaction, which means any adverse event caused by a drug.

8.3 Immune-mediated AE

Anti-PD-1 antibody can result in some immune-mediated adverse events probably due to T cell activation and proliferation. The immune-mediated AE is defined as an adverse event of unknown etiology associated with drug exposure and consistent with an immune phenomenon, and after ruling out neoplastic, infectious, metabolic, toxin or other etiologic causes.

Once the immune-mediated adverse reaction is noted, appropriate work-up should be performed, and steroid therapy may be considered if clinically necessary. However, the patient must be informed that the clinical anti-tumor responses have been maintained in patients treated with corticosteroids and discontinued from anti-PD-1 antibody. The skin rash/toxicity should be treatment as ESMO Clinical Practice Guidelines, 2017.

8.4 Manganese-related AE

Manganese is suggested as a bridge between the innate and adaptive immune

reaction. The immune-mediated AE may be also observed after the Manganese administration. Once the immune-mediated adverse reaction is noted, appropriate work-up should be performed, and the usage of Manganese Chloride should be discontinued.

The overdose of Manganese has been reported to cause a neurologic disorder, just like the Parkinsonian Syndrome. The blood concentration of Manganese must be monitored on time. Once the Manganese is overdosed and/or the neurologic AE appreas, Manganese Chloride must be discontinued.

8.5 Serious AE (SAE)

A SAE is an AE occurring during any study phase that meet the following criteria:

1) Results in death;

2) Is immediately life-threatening, which means the patients lie in risk of death from the AEs as it occurred or it is suspected that use or continued use of the drug would results in patients' death;

3) Requires in-patient hospitalization or prolongation of existing hospital;

4) Results in persistent or significantly disability/incapacity or disruption of the ability to conduct normal life functions;

5) Is a congenital abnormality or birth defect;

6) Is an important medical event that might need medical intervention to prevent one of the outcomes listed above.

8.5 Grading and recording of AEs

AEs will be collected from time of receiving the products throughout the treatment period and including the safety follow-up period (3 months after last dose).

After this study ends, there might be some patients remaining on study treatment. For these patients will continue to collect information about AEs.

Any AE that is unresolved will be followed up by the investigators for as long as

medically indicated. If any investigator learns of any SAEs, including death, at any time, and considers there is a reasonable possibility that the events, the results need document.

All events with an assigned CTCAE grades use the grading scales in the current National Cancer Institute CTCAE, version 5.0. For those events without assigned CTCAE grades, the severity of mild, moderate, severe and life-threatening, corresponding to Grades 1 - 5, as flowing:

- Grade 1, Mild; asymptomatic or mild symptoms; clinical or diagnostic observations only; intervention not indicated.
- Grade 2, Moderate; minimal, local or noninvasive intervention indicated; limiting age- appropriate instrumental Activities of Daily Living (ADL) (refer to preparing meals, shopping for groceries or clothes, using the telephone, managing money, etc).
- Grade 3, Severe or medically significant but not immediately life-threatening; hospitalization or prolongation of hospitalization indicated; disabling; limiting self-care ADL (refer to bathing, dressing and undressing, feeding self, using the toilet, taking medications, and not bedridden).
- Grade 4, Life-threatening consequences; urgent intervention indicated.
- Grade 5, Death related to AE.

The following variables will be collected for each AE:

AEs	AE;<br>The data when the AE started and stopped;<br>Changes in CTCAE grade;<br>The serious grade of the AE;<br>Action taken with regard to investigational product;<br>Outcome;<br>Treatment measures.
SAEs	Date AE met criteria for SAE;<br>The cause of serious AE;<br>Date of hospitalization;<br>Date of discharge;<br>Causality assessment in relation to study procedures;<br>Description of AE.

8.6 The report of deaths

All deaths during the study or within the follow-up period should be reported. If

death is directly due to disease progression, it should be communicated to the study monitor at the next monitoring visit and should be documented in the case report form, but should not be reported as a SAE in the study; When death is not clearly due to disease progression or the disease under study the AE causing the death should be reported to the study monitor as an SAE within 24 hours. The report should assign the primary cause of death with any contributory causes.

8.7 Management of the treatment related toxicities

If patients experience unacceptable toxicity, the patient should be withdrawn from the study and observed until the toxicity was resolved.

If patients show ≥ grade 3-toxicity, and the investigators consider the AE of concern to be specifically associated with investigational drugs, dosing will be interrupted and supportive therapy administered as required in accordance with guidelines.

If the toxicity resolves or reverts to ≤ grade 2 within 21 days, treatment with investigational drugs may be restarted at the same dose.

9. Statistical analyses

9.1 Statistical considerations

The detailed summary of the data collected in this study and the statistical analysis method will be prepared prior to first subject enrolled and recorded in the statistical analysis plan (SAP). If any change to the study protocol is judged by the principal investigator to have an important effect on the statistical analysis plan, the SAP needs to be re-modified as to keep consistency with the study protocol.

The primary objective of this trial is to determine safety and mode of delivery of mode with a minimal follow-up of 6 months, the data cutoff will take place 6 months after the last subject enrolled. The secondary objective is further analysis of the efficacy parameters, including ORR and DCR, and the q3w pharmacokinetic profile

of Manganese will be reported.

All the subjects who have taken at least one dose of the investigational products will be considered as the **safety- assessable population**. All the subjects enrolled who have taken at least two dose of the investigational product and received at least one evaluable post-treatment tumor scan will be accepted as the **efficacy-evaluable population**.

9.2 Sample size determination

The sample size is based on clinical and regulatory considerations and has no formal statistical basis. Three to 6 patients will be enrolled per dose level. The exact sample size cannot be pre-defined, as the number of patients treated will depend on the toxicity observed. Patients enrolled and who discontinue from the study during the first treatment cycle for reasons other than DLT may be replaced.

9.3 Outcome assessment

1) Objective Response Rate (ORR)

ORR is defined as the proportion of patients with at least one visit response of CR or PR that is confirmed at least 6 weeks later. Data obtained up until progression, or last evaluable assessment in the absence of progression, will be included in the assessment of objective response. However, any CR or PR that occurred after a further anticancer therapy will not be included in ORR analysis.

2) Disease Control Rate (DCR)

DCR is defined as the proportion of patients with at least one visit response of CR, PR or SD that is confirmed at least 6 weeks later. Data obtained up until progression, or last evaluable assessment in the absence of progression, will be included in the assessment. However, any response that occurred after a further anticancer therapy will not be included in DCR analysis.

3) Tumor Shrinkage

Tumor shrinkage is assessed using lymphoma response. The SPD (sum of the

product of the diameters) change and percentage change from baseline in sum of tumor size at each assessment will be calculated. The best change in tumor size will include all assessment prior to progression or start of subsequent anticancer therapy.

9.4 Safety assessment

Adverse events will be listed individually by patient. Any AE occurring within 3 months of discontinuation of investigational product will be including in the AE summaries. For change from baseline summaries for vital signs, laboratory data and physical examination, the baseline value will be the latest result obtained prior to the start of investigational product. The denominator in vital signs data should include only those patients with recorded data, and in laboratory summaries will only include evaluable patients, in other words those who had sufficient data to have the possibility of an abnormality.

9.5 Blood Manganese concentration assessment

The blood samples were collected on the first day prior administration of each cycle in heparinized tube. 20 μL sample centrifuged at 4000 rpm for 1 min after standing 15 min for the trace element activation. The concentration of free blood Mn^{2+} was detected with High Precision Trace Element Analyzer (AASA medical technology Co., Wuhan, China), which was approved by the National Medical Products Administration of China (2013-2401836). The normal range of free blood Mn2+ was 0.029-1.2 μmol/L.

9.6 Peripheral blood immune cell and serum cytokine/chemokine assessment

Blood samples will be collected for peripheral immune cell assessment on the first day prior administration of each cycle. The peripheral blood was collected in sodium heparin anticoagulant vacutainer tubes. Briefly, 100 μl of the anticoagulant peripheral blood was incubated with antibodies specific to cell-surface antigens expressed on DC, NK, macrophage, neutrophil and T lymphocytes. After red blood

cell lysis and washing, the cells were detected on a BD FACSCalibur flow cytometer (BD Biosciences). The antibodies were used to detect surface marker expression and obtained from BD Biosciences. For the intracellular cytokine expression, blood cells were stimulated with T cell stimulation cocktail (including PMA, Ionomycin and transport inhibitors, eBioscience, 00-4975-93) for 4 h of incubation, and cells were stained with anti-CD3 and permeabilized before the addition of anti-IFN-γ (554700). Stained cells were detected by flow cytometry using FACSCalibur flow cytometer (BD Biosciences) to collect a minimum of 10,000 CD3+ lymphocytes.

Serum samples will be collected for cytokines and chemokines analysis on the first day prior administration of both the first and third treatment cycles. The detected cytokines and chemokines include IL-1β、2、 4、6、8 (CXCL8)、10、1、17A, IFN-α、β、γ, TNF-α, soluble Fas (sFas), soluble FasL (sFasL), granzyme A, granzyme B, perforin, granulysin, IP-10 and GM-CSF. The analysis was performed using 200 μl serum of each sample via LEGENDplex™ bead-based immunoassays (Biolegend, LEGENDplex™ Human CD8/NK Panel [740267], LEGENDplex™ Human anti-virus response Panel [740349]).

9.7 Data analysis

Appropriate descriptive statistics will be used for all variables. Continuous variables will be summarized by mean, standard deviation, median, maximum and minimum. Categorical variable will be summarized by frequency counts and percentages for each category. Unless otherwise stated, percentages will be calculated out of the full analysis set.

ORR and DCR will be presented together with two-sided 95% exact confidence interval (CI), which is calculated using the Wilson method. Summaries of the number and percentage of patients with best response in each of the follow categories will be summarized: complete remission (CR), partial response (PR), stable disease (SD), progressive disease (PD) and not evaluable (NE).

The best absolute change in target lesion tumor size from baseline and

percentage change in target lesion tumor size from baseline will be summarized using descriptive statistics and presented at each time point.

List of abbreviations

Abbreviation	Full name
ADL	Activities of Daily Living
AE	adverse event
ALT	alanine aminotransferase
ANC	absolute neutrophil count
ASCT	autologous stem-cell transplantation
AST	aspartate aminotransferase
BUN	blood urea nitrogen
CAR-T	Chimeric antigen receptor T cell
CI	confidence interval
CR	complete response
CT	computed tomography
CTCAE	Common Terminology Criteria for Adverse Events
CTLA-4	Cytotoxic T lymphocyte-associated antigen 4
DCR	Disease control rate
DLT	dose-limiting toxicity
ECG	Electrocardiogram
ECHO	Echocardiogram
ECOG	Eastern Cooperative Oncology Group
ESMO	European Society for Medical Oncology
FACS	fluorescence activated cell sorting
FDA	Food and Drug Administration
G-CSF	granulocyte colony stimulating factor
Hb	hemoglobin
HBV	hepatitis B virus
HCG	human chorionic gonadotropin
HCV	hepatitis C virus
HIV	human immunodeficiency virus
HL	Hodgkin’s lymphoma

HPR	horseradish peroxidase
IRB	Institutional Review Board
IV	intravenous
LDH	lactate dehydrogenase
MTD	Maximum Tolerated Dose
MRI	Magnetic Resonance Imaging
NCI	National Cancer Institute
NE	not evaluable
ORR	objective response
PD	progression disease
PD-1	Programmed death-1
PD-L1	Programmed death ligand 1
PD-L2	Programmed death ligand 2
PR	partial response
RECIST	Response Evaluation Criteria in Solid Tumors
RP2D	Phase 2 Dose
SAE	serious adverse event
SAP	statistical analysis plan
SD	stable disease
SOPs	standard operating procedures
SPD	sum of the product of the diameters
ULN	upper limit of normal
WBC	white blood cell

Reference

1. Ricklin D, Hajishengallis G, Yang K, Lambris JD. Complement: a key system for immune surveillance and homeostasis. *Nat Immunol* 2010;11: 785-97.
2. Corthay A. Does the immune system naturally protect against cancer? *Front Immunol* 2014;5: 197.
3. Lohmueller J, Finn OJ. Current modalities in cancer immunotherapy: Immunomodulatory antibodies, CARs and vaccines. *Pharmacol Ther* 2017;178: 31-47.
4. Zou W, Wolchok JD, Chen L. PD-L1 (B7-H1) and PD-1 pathway blockade for cancer therapy: Mechanisms, response biomarkers, and combinations. *Sci Transl Med* 2016;8: 328rv4.
5. Chen L, Han X. Anti-PD-1/PD-L1 therapy of human cancer: past, present, and future. *J Clin Invest* 2015;125: 3384-91.
6. Topalian SL, Drake CG, Pardoll DM. Targeting the PD-1/B7-H1(PD-L1) pathway to activate anti-tumor immunity. *Curr Opin Immunol* 2012;24: 207-12.
7. Escors D, Gato-Canas M, Zuazo M, Arasanz H, Garcia-Granda MJ, Vera R, Kochan G. The intracellular signalosome of PD-L1 in cancer cells. *Signal Transduct Target Ther* 2018;3: 26.
8. Gato-Canas M, Zuazo M, Arasanz H, Ibanez-Vea M, Lorenzo L, Fernandez-Hinojal G, Vera R, Smerdou C, Martisova E, Arozarena I, Wellbrock C, Llopiz D, Ruiz M, Sarobe P, Breckpot K, Kochan G, Escors D. PDL1 Signals through Conserved Sequence Motifs to Overcome Interferon-Mediated Cytotoxicity. *Cell Rep* 2017;20: 1818-29.
9. Dong H, Strome SE, Salomao DR, Tamura H, Hirano F, Flies DB, Roche PC, Lu J, Zhu G, Tamada K, Lennon VA, Celis E, Chen L. Tumor-associated B7-H1 promotes T-cell apoptosis: a potential mechanism of immune evasion. *Nat Med* 2002;8: 793-800.
10. Reiss KA, Forde PM, Brahmer JR. Harnessing the power of the immune system

via blockade of PD-1 and PD-L1: a promising new anticancer strategy. *Immunotherapy* 2014;6: 459-75.

11. Keane C, Vari F, Hertzberg M, Cao KA, Green MR, Han E, Seymour JF, Hicks RJ, Gill D, Crooks P, Gould C, Jones K, Griffiths LR, Talaulikar D, Jain S, Tobin J, Gandhi MK. Ratios of T-cell immune effectors and checkpoint molecules as prognostic biomarkers in diffuse large B-cell lymphoma: a population-based study. *Lancet Haematol* 2015;2: e445-55.
12. Ghoneim HE, Fan Y, Moustaki A, Abdelsamed HA, Dash P, Dogra P, Carter R, Awad W, Neale G, Thomas PG, Youngblood B. De Novo Epigenetic Programs Inhibit PD-1 Blockade-Mediated T Cell Rejuvenation. *Cell* 2017;170: 142-57 e19.
13. Motzer RJ, Escudier B, McDermott DF, George S, Hammers HJ, Srinivas S, Tykodi SS, Sosman JA, Procopio G, Plimack ER, Castellano D, Choueiri TK, Gurney H, Donskov F, Bono P, Wagstaff J, Gauler TC, Ueda T, Tomita Y, Schutz FA, Kollmannsberger C, Larkin J, Ravaud A, Simon JS, Xu LA, Waxman IM, Sharma P, CheckMate I. Nivolumab versus Everolimus in Advanced Renal-Cell Carcinoma. *N Engl J Med* 2015;373: 1803-13.
14. Chen DS, Mellman I. Oncology meets immunology: the cancer-immunity cycle. *Immunity* 2013;39: 1-10.
15. Chen DS, Mellman I. Elements of cancer immunity and the cancer-immune set point. *Nature* 2017;541: 321-30.
16. Kim JM, Chen DS. Immune escape to PD-L1/PD-1 blockade: seven steps to success (or failure). *Ann Oncol* 2016;27: 1492-504.
17. Liu Q, Das M, Liu Y, Huang L. Targeted drug delivery to melanoma. *Adv Drug Deliv Rev* 2018;127: 208-21.
18. Gubin MM, Zhang X, Schuster H, Caron E, Ward JP, Noguchi T, Ivanova Y, Hundal J, Arthur CD, Krebber WJ, Mulder GE, Toebes M, Vesely MD, Lam SS, Korman AJ, Allison JP, Freeman GJ, Sharpe AH, Pearce EL, Schumacher TN, Aebersold R, Rammensee HG, Melief CJ, Mardis ER, Gillanders WE, Artyomov MN, Schreiber RD. Checkpoint blockade cancer immunotherapy targets tumour-specific mutant antigens. *Nature* 2014;515: 577-81.

19. Sharma P, Hu-Lieskovan S, Wargo JA, Ribas A. Primary, Adaptive, and Acquired Resistance to Cancer Immunotherapy. *Cell* 2017;168: 707-23.

20. Pfirschke C, Engblom C, Rickelt S, Cortez-Retamozo V, Garris C, Pucci F, Yamazaki T, Poirier-Colame V, Newton A, Redouane Y, Lin YJ, Wojtkiewicz G, Iwamoto Y, Mino-Kenudson M, Huynh TG, Hynes RO, Freeman GJ, Kroemer G, Zitvogel L, Weissleder R, Pittet MJ. Immunogenic Chemotherapy Sensitizes Tumors to Checkpoint Blockade Therapy. *Immunity* 2016;44: 343-54.

21. Fan W, Yung B, Huang P, Chen X. Nanotechnology for Multimodal Synergistic Cancer Therapy. *Chem Rev* 2017;117: 13566-638.

22. Castano AP, Mroz P, Hamblin MR. Photodynamic therapy and anti-tumour immunity. *Nat Rev Cancer* 2006;6: 535-45.

23. Kroemer G, Galluzzi L, Kepp O, Zitvogel L. Immunogenic cell death in cancer therapy. *Annu Rev Immunol* 2013;31: 51-72.

24. Wang C, Wang J, Zhang X, Yu S, Wen D, Hu Q, Ye Y, Bomba H, Hu X, Liu Z, Dotti G, Gu Z. In situ formed reactive oxygen species-responsive scaffold with gemcitabine and checkpoint inhibitor for combination therapy. *Sci Transl Med* 2018;10.

25. Liu WM, Fowler DW, Smith P, Dalgleish AG. Pre-treatment with chemotherapy can enhance the antigenicity and immunogenicity of tumours by promoting adaptive immune responses. *Br J Cancer* 2010;102: 115-23.

26. Woo SR, Corrales L, Gajewski TF. The STING pathway and the T cell-inflamed tumor microenvironment. *Trends Immunol* 2015;36: 250-6.

27. Woo SR, Fuertes MB, Corrales L, Spranger S, Furdyna MJ, Leung MY, Duggan R, Wang Y, Barber GN, Fitzgerald KA, Alegre ML, Gajewski TF. STING-dependent cytosolic DNA sensing mediates innate immune recognition of immunogenic tumors. *Immunity* 2014;41: 830-42.

28. Deng L, Liang H, Xu M, Yang X, Burnette B, Arina A, Li XD, Mauceri H, Beckett M, Darga T, Huang X, Gajewski TF, Chen ZJ, Fu YX, Weichselbaum RR. STING-Dependent Cytosolic DNA Sensing Promotes Radiation-Induced Type I Interferon-Dependent Antitumor Immunity in Immunogenic Tumors. *Immunity*

2014;41: 843-52.

29. Wang C, Guan Y, Lv M, Zhang R, Guo Z, Wei X, Du X, Yang J, Li T, Wan Y, Su X, Huang X, Jiang Z. Manganese Increases the Sensitivity of the cGAS-STING Pathway for Double-Stranded DNA and Is Required for the Host Defense against DNA Viruses. *Immunity* 2018;48: 675-87 e7.

Appendix 1. Time and event schema

	Baseline	Each cycle	Follow-up									
Visit name	V0		V7	V8	V9	V10	V11	V12	V13	V14	V15	V16
Week	-2 to -1		36±3	48±3	72±3	96±3	120±7	144±7	168±7	192±7	216±7	240±7
Month			9	12	18	24	30	36	42	48	54	60
Inform consent	√											
Demography	√											
Clinical history	√											
Pathologic result conformation	√											
Signs of life	√	√	√	√	√	√	√	√	√	√	√	√
Physical examination	√	√	√	√	√	√	√	√	√	√	√	√
General information	√	√	√	√	√	√	√	√	√	√	√	√
IHC	√		√									
ECOG	√	√	√	√	√	√	√	√	√	√	√	√
Blood cell count	√											
Urine routine	√	√	√	√	√	√	√	√	√	√	√	√
Stool routine	√	√	√	√	√	√	√	√	√	√	√	√
Renal/Liver function	√	√	√	√	√	√	√	√	√	√	√	√
HCG	√	√	√	√	√	√	√	√	√	√	√	√
Coagulation function	√	√	√	√	√	√	√	√	√	√	√	√
Infectious disease screening	√	√	√	√	√	√	√	√	√	√	√	√
Immune function	√	√	√	√	√	√	√	√	√	√	√	√
Laboratory assessments	√	√	√	√	√	√	√	√	√	√	√	√
ECG	√	√	√	√	√	√	√	√	√	√	√	√
ECHO	√	√	√	√	√	√	√	√	√	√	√	√
CT	√	Every 2 cycles	√	√	√	√	√	√	√	√	√	√
MRI	√	Every 2 cycles	√		√		√		√		√	
PMBC collection	√	Before each cycle										
Serum collection	√	Before each cycle										
AE evaluation		√	√	√								

Appendix 2. Tumor response criteria

1. Time point response: patients with target (+/–non-target) disease

Target lesions	Non-target lesions	New lesions	Overall response
CR	CR	No	CR
CR	Non-CR/non-PD	No	PR
CR	Not evaluated	No	PR
PR	Non-PD or not all evaluated	No	PR
SD	Non-PD or not all evaluated	No	SD
Not all evaluated	Non-PD	No	NE
PD	Any	Yes or No	PD
Any	PD	Yes or No	PD
Any	Any	Yes	PD

CR = complete response, PR = partial response, SD = stable disease, PD = progressive disease, and NE = inevaluable.

2. Best overall response when confirmation of CR and PR required

Overall response First time point	Overall response Subsequent time point	BEST overall response
CR	CR	CR
CR	PR	SD, PD or PR[a]
CR	SD	SD provided minimum criteria for SD duration met, otherwise, PD
CR	PD	SD provided minimum criteria for SD duration met, otherwise, PD
CR	NE	SD provided minimum criteria for SD duration met, otherwise NE
PR	CR	PR
PR	PR	PR
PR	SD	SD
PR	PD	SD provided minimum criteria for SD duration met, otherwise, PD
PR	NE	SD provided minimum criteria for SD duration met, otherwise NE
NE	NE	NE

CR = complete response, PR = partial response, SD = stable disease, PD = progressive disease, and NE = inevaluable.

a If a CR is *truly* met at first time point, then any disease seen at a subsequent time point, even disease meeting PR criteria relative to baseline, makes the disease PD at that point (since disease must have reappeared after CR). Best response would depend on whether minimum duration for SD was met. However, sometimes 'CR' may be claimed when subsequent scans suggest small lesions were likely still present and in fact the patient had PR, not CR at the first time point. Under these circumstances, the original CR should be changed to PR and the best response is PR.

3. Measured dominant lesions

Up to 3 of the target lesions selected to be clearly measurable in 2 diameters. Tumor assessments were performed at the baseline, every 6 weeks by CT and/or MRI every 6 weeks. All CT and MRI scans were reviewed and scored by the same radiologist.

4. Non-measured lesions

Any disease not selected as measured, dominant disease and truly assessable disease should be considered not measured. These sites do not meet the requirements for measurability, but are still considered abnormal. As well as truly assessable disease which is any site of suspected disease that would be difficult to follow quantitiatively with measurement, including pleural effusions, ascites, bone lesions, leptomeningeal disease, abdominal masses and other lesions that cannot be confirmed and followed by imaging.

Appendix 3. Common terminology criteria for adverse events (CTCAE), v5.0

The descriptions and grading scales found in the revised NCI Common Terminology Criteria for Adverse Events (CTCAE) version 5.0 will be utilized for adverse event reporting. (http://ctep.cancer.gov/reporting/ctc.html)

Appendix 4. ECOG performance status scale

Grade	Description
**0**	Normal activity. Fully active, able to carry on all pre-disease performance without restriction.
**1**	Symptoms, but ambulatory. Restricted in physically strenuous activity, but ambulatory and able to carry out work of a light or sedentary nature (e.g., light housework, office work).
**2**	In bed < 50% of the time. Ambulatory and capable of all self-care, but unable to carry out any work activities. Up and about more than 50% of waking hours.
**3**	In bed > 50% of the time. Capable of only limited self-care, confined to bed or chair more than 50% of waking hours.
**4**	100% bedridden. Completely disabled. Cannot carry on any self-care. Totally confined to bed or chair.
**5**	Dead.

Appendix 5. New York heart association (NYHA) classification

Class I: patients with no limitation of activities; they suffer no symptoms from ordinary activities.

Class II: patients with slight, mild limitation of activity; they are comfortable with rest or with mild exertion.

Class III: patients with marked limitation of activity; they are comfortable only at rest.

Class IV: patients who should be at complete rest, confined to bed or chair; any physical activity brings on discomfort and symptoms occur at rest.
