# Supplementary material for: Manganese is critical for antitumor immune responses via cGAS-STING and improves the efficacy of clinical immunotherapy
Source: Cell Res. 2020 Aug 24;30(11):966–79. doi: 10.1038/s41422-020-00395-4 (PMC7785004; doi:10.1038/s41422-020-00395-4)
Supplement: Supplementary file 2 — Supplementary information, Fig. S1 [file 41422_2020_395_MOESM2_ESM.pdf]

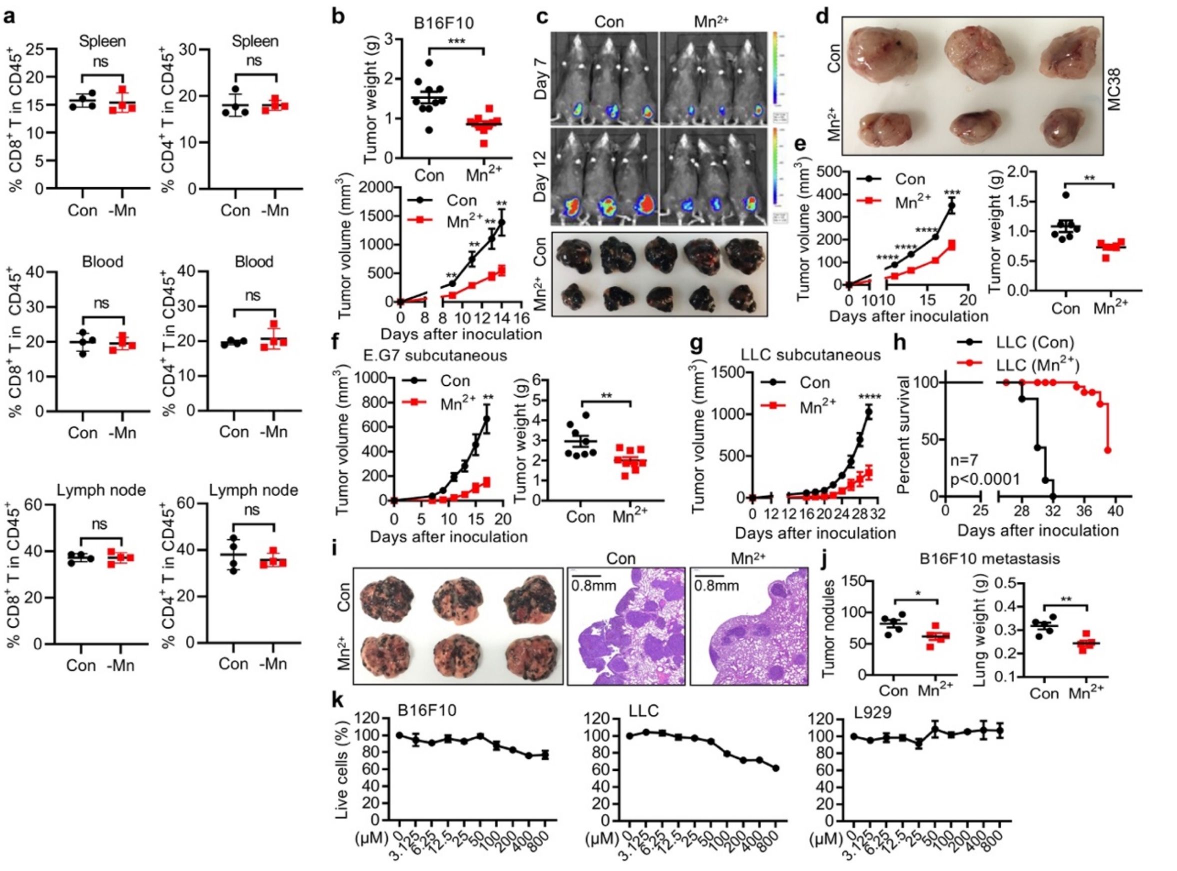

Supplementary Fig. 1 Mn2+ promotes antitumor immune responses. a Frequency of CD8+ T and CD4+ T cells in spleen, blood and inguinal lymph node from control (Con) and Mn-insufficient mice (-Mn). **b** Tumor weights and tumor sizes of B16F10 tumors from the wild-type C57BL/6 (WT) mice treated with 5 mg/kg $MnCl_2$ intranasally (i.n.) with saline as control (n=10) once every two days after injection of 5×10^5 B16F10 subcutaneously (s.c.), tumors were dissected and assessed at day 14 after inoculation. **c** Representative images (top) of *in vivo* bioluminescence of tumors in the right groin of WT mice at day 7 and day 12, mice (n=5 per group) were treated with saline or 5 mg/kg $MnCl_2$ i.n. once every two days after subcutaneous injection of 5×10^5 B16F10 cells. These mice were sacrificed at day 14, tumors were dissected and photographed (bottom). **d** Representative images of MC38 tumors from WT mice treated with 5 mg/kg $MnCl_2$ intravenously (i.v.) with PBS as control (n=7) once every two days after subcutaneous injection of 1×10^6 MC38 cells, tumors were dissected and assessed at day 18 after inoculation. **e** Tumor weights and tumor sizes of MC38 tumors from WT mice as in (**d**). **f** Tumor sizes in WT mice treated with saline or 5 mg/kg $MnCl_2$ i.n. once every two days after subcutaneous injection of 1×10^6 E.G7 cells on day 0 (n=5 per group, left), tumors were dissected and weighted at day 18 after inoculation (Con, n=8; Mn, n=9, right). **g, h** Tumor size (**g**) and survival (**h**) of WT mice treated with saline or 5 mg/kg $MnCl_2$ i.n. once every two days after subcutaneous injection of 1×10^6 LLC cells on day 0 (n=7 per group). **i, j** Representative images (**i**) and quantification (**j**) of tumor nodules and lung weights of saline or 5 mg/kg $MnCl_2$ treated mice (i.n., n=5 per group) at day 15 after intravenous injection of 2×10^5 B16 cells. **k** MTT staining of B16F10, LLC or L929 treated with the indicated concentrations of $MnCl_2$ for 24 h. Data represent analyses of the indicated n mice per group, mean ± SEM. Data are representative of three independent experiments. ns, not significant; *$p < 0.05$, **$p < 0.01$, ***$p < 0.001$, ****$p < 0.0001$.
