# Supplementary material for: Manganese is critical for antitumor immune responses via cGAS-STING and improves the efficacy of clinical immunotherapy
Source: Cell Res. 2020 Aug 24;30(11):966–79. doi: 10.1038/s41422-020-00395-4 (PMC7785004; doi:10.1038/s41422-020-00395-4)
Supplement: Supplementary file 3 — Supplementary information, Fig. S2 [file 41422_2020_395_MOESM3_ESM.pdf]

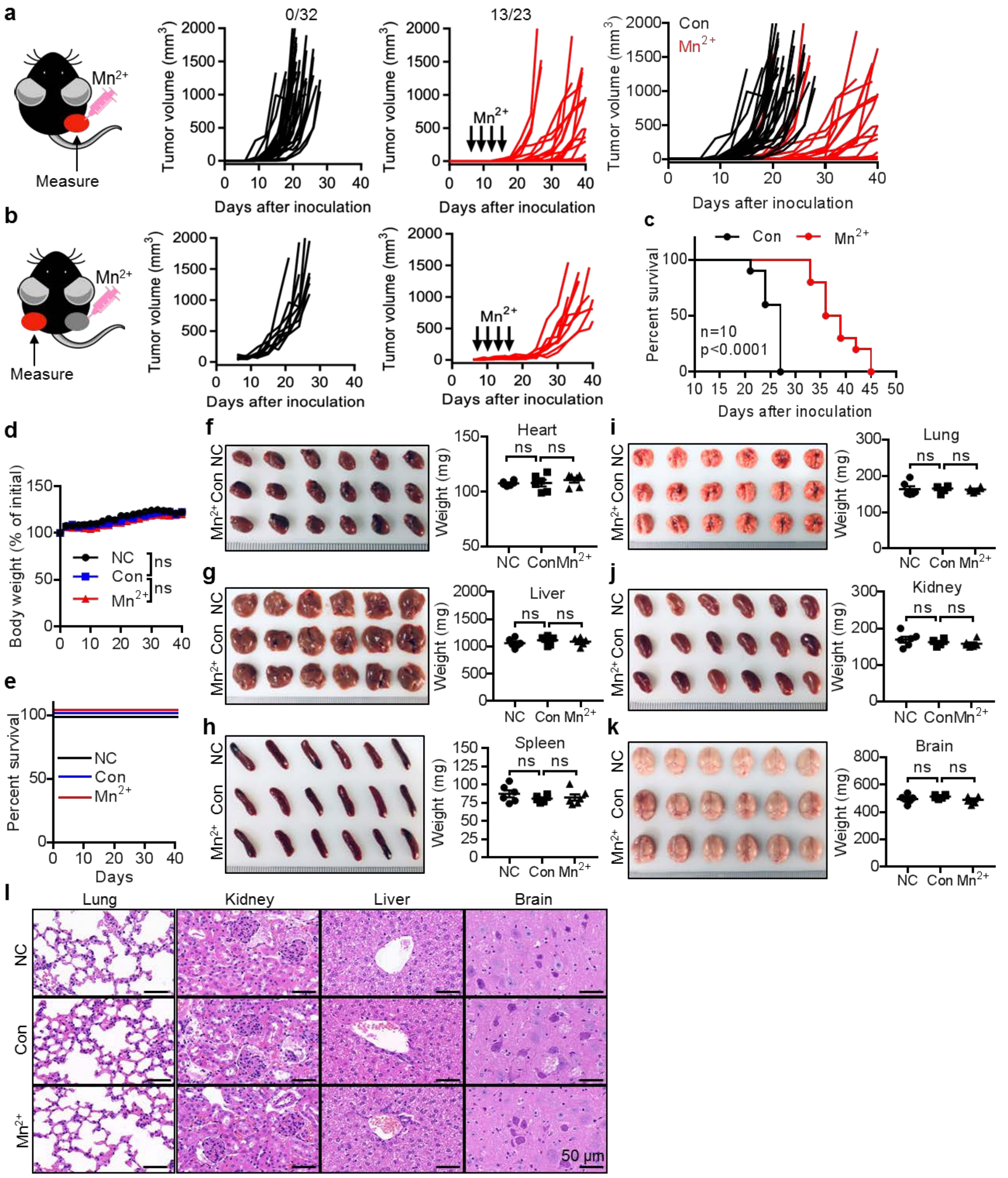

Supplementary Fig. 2 Mn^{2+} is a potent chemotherapeutic agent without detectable toxicity to mice. a The WT mice were treated with saline or 5 mg/kg $MnCl_2$ intratumorally (i.t.) at day 8, 10, 12, 14 after subcutaneous inoculation of 3×10^5 B16-OVA cells in the right groin (Con, n=32; Mn^{2+}, n=23), the sizes of tumor were recorded. No mice (0/32) in the control group and 13 mice (13/23) in Mn^{2+}-treated group survived over 40 days. **b, c** The WT mice were subcutaneously inoculated with 1.5×10^5 B16-OVA cells in both left and right groin respectively, one side of tumors was treated with saline or 5 mg/kg $MnCl_2$ i.t. at day 8, 10, 12, 14 after inoculation (Con, n=10; Mn^{2+}, n=10), the sizes of tumors on the non-treated side were recorded (**b**). Their survival rates were monitored everyday (**c**). **d, e** The WT mice (n=6) were left untreated (NC) or treated with saline (Con, n=6) or 5mg/kg $MnCl_2$ (Mn^{2+}, n=6) i.n. on day 0, 2, 4, 6, 8, 10 and 12. Their body weights (**d**) were monitored every two days and their survival rates (**e**) were monitored every day. **f-k** On day 40, hearts (**f**), livers (**g**), spleens (**h**), lungs (**i**), kidneys (**j**) and brains (**k**) were removed

and organ weights were recorded. **l** HE staining of livers, lungs, kidneys, and brains dissected from (**g, i, j, k**). Data represent analyses of the indicated n mice per group, mean ± SEM. Data are representative of three independent experiments. ns, not significant; ****p <0.0001.
