# Supplementary material for: Manganese is critical for antitumor immune responses via cGAS-STING and improves the efficacy of clinical immunotherapy
Source: Cell Res. 2020 Aug 24;30(11):966–79. doi: 10.1038/s41422-020-00395-4 (PMC7785004; doi:10.1038/s41422-020-00395-4)
Supplement: Supplementary file 4 — Supplementary information, Fig. S3 [file 41422_2020_395_MOESM4_ESM.pdf]

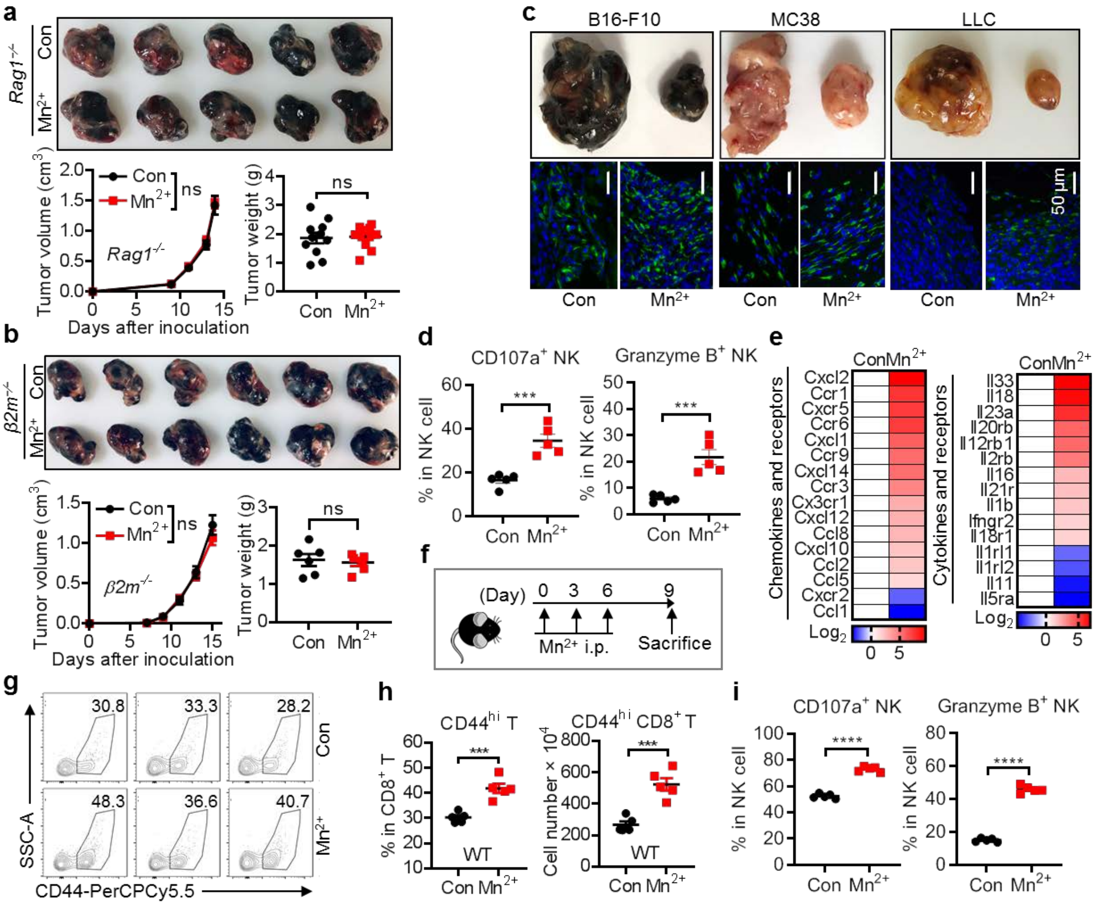

Supplementary Fig. 3 Mn^{2+} stimulates $CD8^+$ T cell and NK cell activation. a Representative images of tumors (top), tumor sizes and tumor weights (bottom) in *Rag1*$^{-/-}$ mice (n=11 per group) treated with saline or 5 mg/kg $MnCl_2$ i.n. at various days as indicated after subcutaneous inoculation of 5 × 10^5 B16F10 cells. **b** Images of tumors (top), tumor sizes and tumor weights (bottom) in *β2m*$^{-/-}$ mice (n=6 per group) treated with saline or 5 mg/kg $MnCl_2$ i.n. at various days as indicated after subcutaneous inoculation of 5 × 10^5 B16F10 cells. **c** Representative images and immunofluorescence slices of tumors in mice subcutaneously inoculated with B16F10 (left), MC38 (middle) or LLC (right). **d** The WT mice were subcutaneously inoculated with 2×10^5 B16F10 cells and treated with saline or 5 mg/kg $MnCl_2$ i.p.. Mice (n=5 per group) were sacrificed on day 16 and tumors were dissected for FACS analysis. The percentage of NK cells expressing CD107a and Granzyme B was assessed. **e** Heatmap of selected genes between $CD8^+$ TILs from saline (Con) and Mn^{2+} treated (i.n.) mice. Heat map was made by calculating log2 ((Mn FPKM)/(Con FPKM)) and values of genes in the control group were normalized to zero. **f-h** Experimental protocol used in (**g**, **h**): WT mice (n=5 per group) were given 5 mg/kg $MnCl_2$ intraperitoneally (i.p.) at the indicated times and sacrificed at day 9. Frequency (**g**) and cell number (**h**) of $CD44^{hi}CD8^+$ T among splenic cells in WT mice (**f**). **i** Quantification of $CD107a^+$ NK and Granzyme B^+ NK in NK cells isolated from mouse spleens treated with 600 μM $MnCl_2$ for 18 h. The data represent analyses of n mice per group, mean ± SEM. Data represent analyses of the indicated n mice per group, mean ± SEM. Data are representative of three independent experiments. ns, not significant; ***p <0.001; ****p <0.0001.
