# Supplementary material for: Manganese is critical for antitumor immune responses via cGAS-STING and improves the efficacy of clinical immunotherapy
Source: Cell Res. 2020 Aug 24;30(11):966–79. doi: 10.1038/s41422-020-00395-4 (PMC7785004; doi:10.1038/s41422-020-00395-4)
Supplement: Supplementary file 5 — Supplementary information, Fig. S4 [file 41422_2020_395_MOESM5_ESM.pdf]

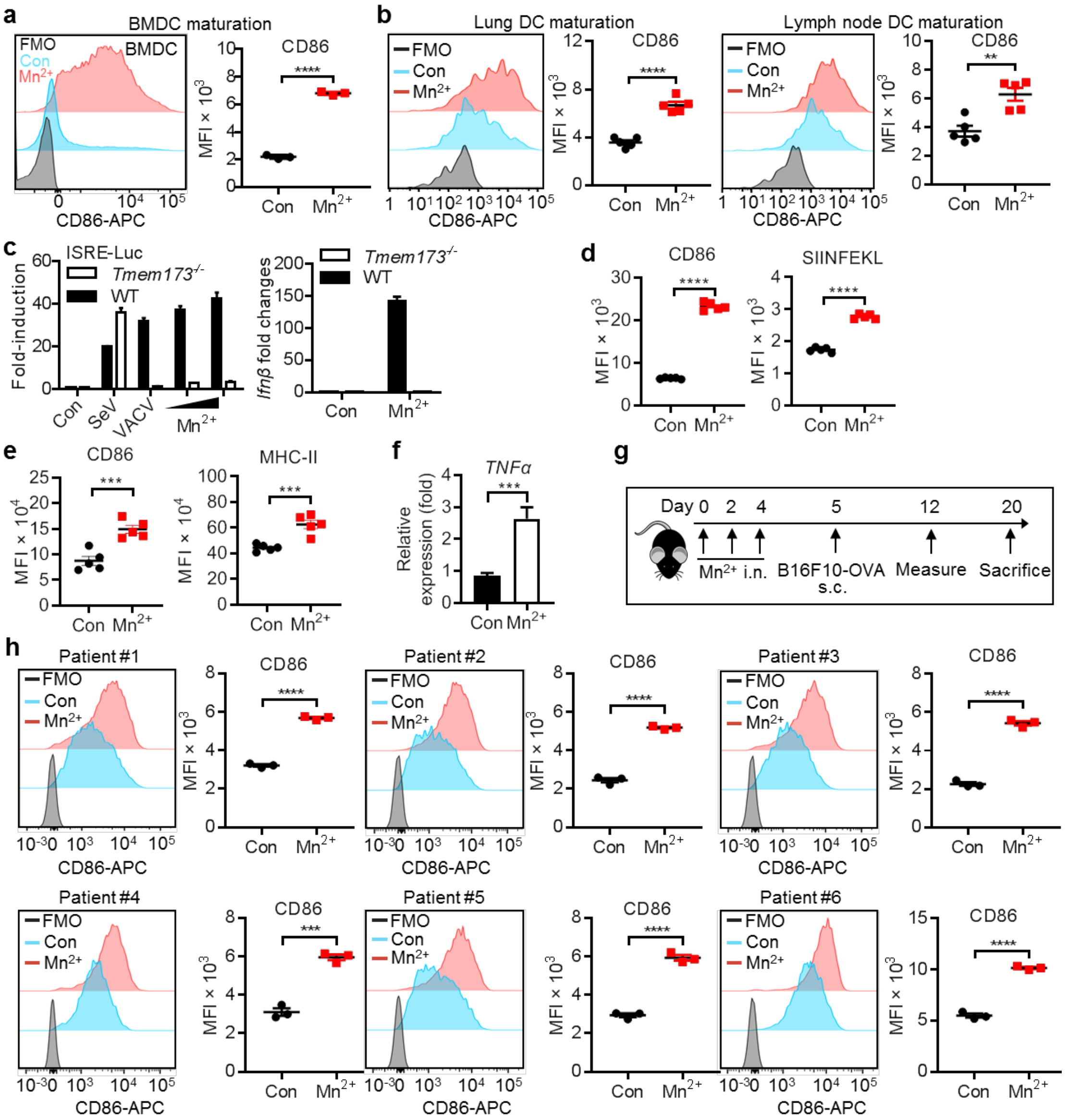

Supplementary Fig. 4 Mn^{2+} promotes DC maturation and antigen presentation. a Mean fluorescent intensity (MFI) of CD86 in BMDCs treated with 400 μM $MnCl_2$ for 18 h. **b** Mean fluorescent intensity (MFI) of CD86 in lung DCs ($MnCl_2$ i.n.) or inguinal lymph node DCs ($MnCl_2$ s.c.) from mice (n=5 per group) treated with 5 mg/kg $MnCl_2$ for 18 h. **c** Type I IFN activity in culture media from the WT or *Tmem173*$^{-/-}$ bone marrow derived macrophages (BMDMs) treated with SeV, VACV, LPS or the indicated concentrations (200 μM and 400 μM) of $MnCl_2$ for 18 h (left). qRT-PCR analysis of *Ifnβ* in BMDM treated with or without $MnCl_2$ (Right). **d** Mean fluorescent intensity (MFI) of CD86 and SIINFEKL in bone marrow-derived macrophage (BMDMs) treated with 400 μM $MnCl_2$ for 18 h. **e** The WT mice were subcutaneously inoculated with $2{\times}10^5$ B16F10 cells and treated with saline or 5 mg/kg $MnCl_2$ i.p.. Mice (n=5 per group) were sacrificed on day 16 and tumors were dissected for FACS analysis. The expression of CD86 and MHC-II on tumor-infiltrating macrophages was quantified. **f** qRT-PCR analysis of *Tnfα* in alveolar macrophages from mice treated with saline or 5 mg/kg $MnCl_2$ i.n. for 18 h. **g** Experimental protocol used in Fig. 3h, i. **h** Mean fluorescent intensity (MFI) of CD86 in DCs of PBMCs from various types of cancer patients (See details in supplementary Table 3) treated with

100 μM $MnCl_2$ for 18 h. Representative results were shown. FMO: Flow Minus One of CD86. Data are represented as mean ± SEM. ** $p < 0.01$; ***$p < 0.001$; ****$p < 0.0001$.
