# Supplementary material for: Manganese is critical for antitumor immune responses via cGAS-STING and improves the efficacy of clinical immunotherapy
Source: Cell Res. 2020 Aug 24;30(11):966–79. doi: 10.1038/s41422-020-00395-4 (PMC7785004; doi:10.1038/s41422-020-00395-4)
Supplement: Supplementary file 6 — Supplementary information, Fig. S5 [file 41422_2020_395_MOESM6_ESM.pdf]

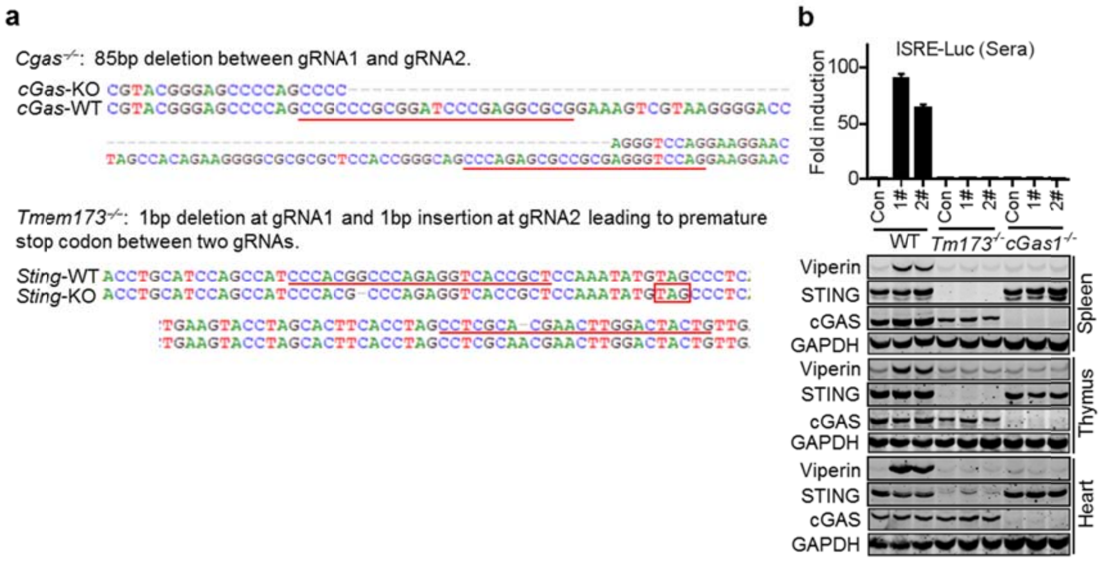

Supplementary Fig. 5 The cGAS-STING pathway is required for Mn^{2+}-mediated antitumor immune responses. a Genotyping of gene-deficient mice in C57BL/6 background. **b** Type I IFN activity in the sera and ISG expression of the indicated organs in $MnCl_2$ treated (i.v.) WT mice and the indicated gene-deficient mice (n=3 per genotype) for 18 h. Data represent analyses of n mice per group, mean ± SEM.
