# Supplementary material for: Manganese is critical for antitumor immune responses via cGAS-STING and improves the efficacy of clinical immunotherapy
Source: Cell Res. 2020 Aug 24;30(11):966–79. doi: 10.1038/s41422-020-00395-4 (PMC7785004; doi:10.1038/s41422-020-00395-4)
Supplement: Supplementary file 7 — Supplementary information, Fig. S6 [file 41422_2020_395_MOESM7_ESM.pdf]

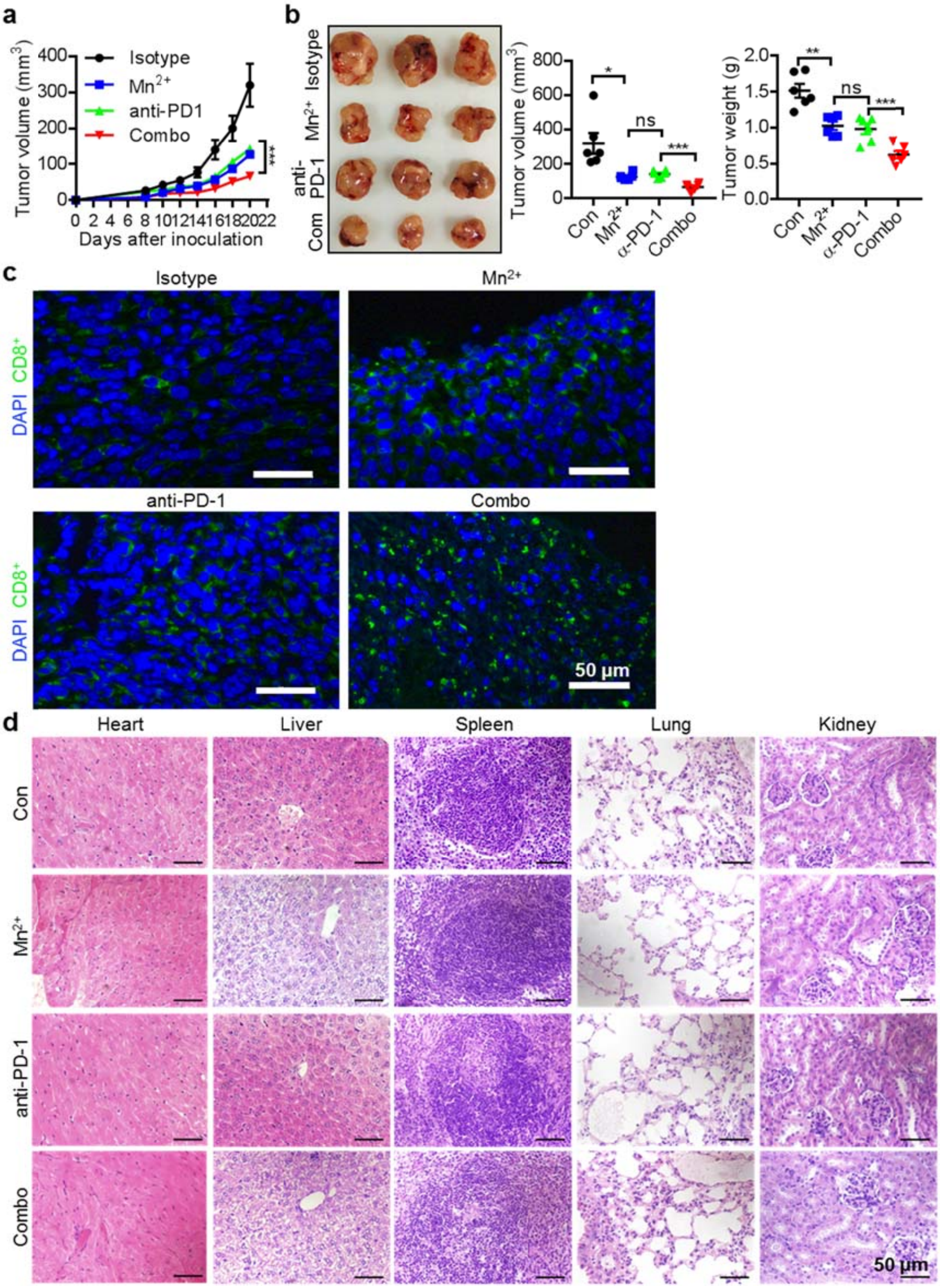

Supplementary Fig. 6 Mn^{2+} boosts antitumor immunotherapy in mice. a Tumor sizes of subcutaneous MC38 implants in mice treated with the isotype antibody (200 μg/mouse i.p.), $MnCl_2$ (5 mg/kg i.v.), anti-PD-1 antibody (200 μg/mouse i.p.) or $MnCl_2$ plus anti-PD-1 antibody (n=6 per group). **b** Representative images (left), tumor sizes (middle) and tumor weights (right) of subcutaneous MC38 implants in mice as in (**a**). **c** MC38 tumors from (**b**) were stained with anti-CD8-FITC and counterstained with DAPI. Scale bar, 50 μm. **d** The indicated organs from

B16F10 tumor-bearing mice treated with $MnCl_2$ (5 mg/kg i.p.), anti-PD-1 antibody (200 µg/mouse i.p.) or $MnCl_2$ plus anti-PD-1 antibody (Combo, n=5 per group) were examined by H & E staining at day 16. Data represent analyses of the indicated n mice per group, mean ± SEM. Data are representative of three independent experiments. ns, not significant, $p>0.05$; $*p < 0.05$; $**p < 0.01$; $***p < 0.001$.
