# Supplementary material for: Manganese is critical for antitumor immune responses via cGAS-STING and improves the efficacy of clinical immunotherapy
Source: Cell Res. 2020 Aug 24;30(11):966–79. doi: 10.1038/s41422-020-00395-4 (PMC7785004; doi:10.1038/s41422-020-00395-4)
Supplement: Supplementary file 8 — Supplementary information, Fig. S7 [file 41422_2020_395_MOESM8_ESM.pdf]

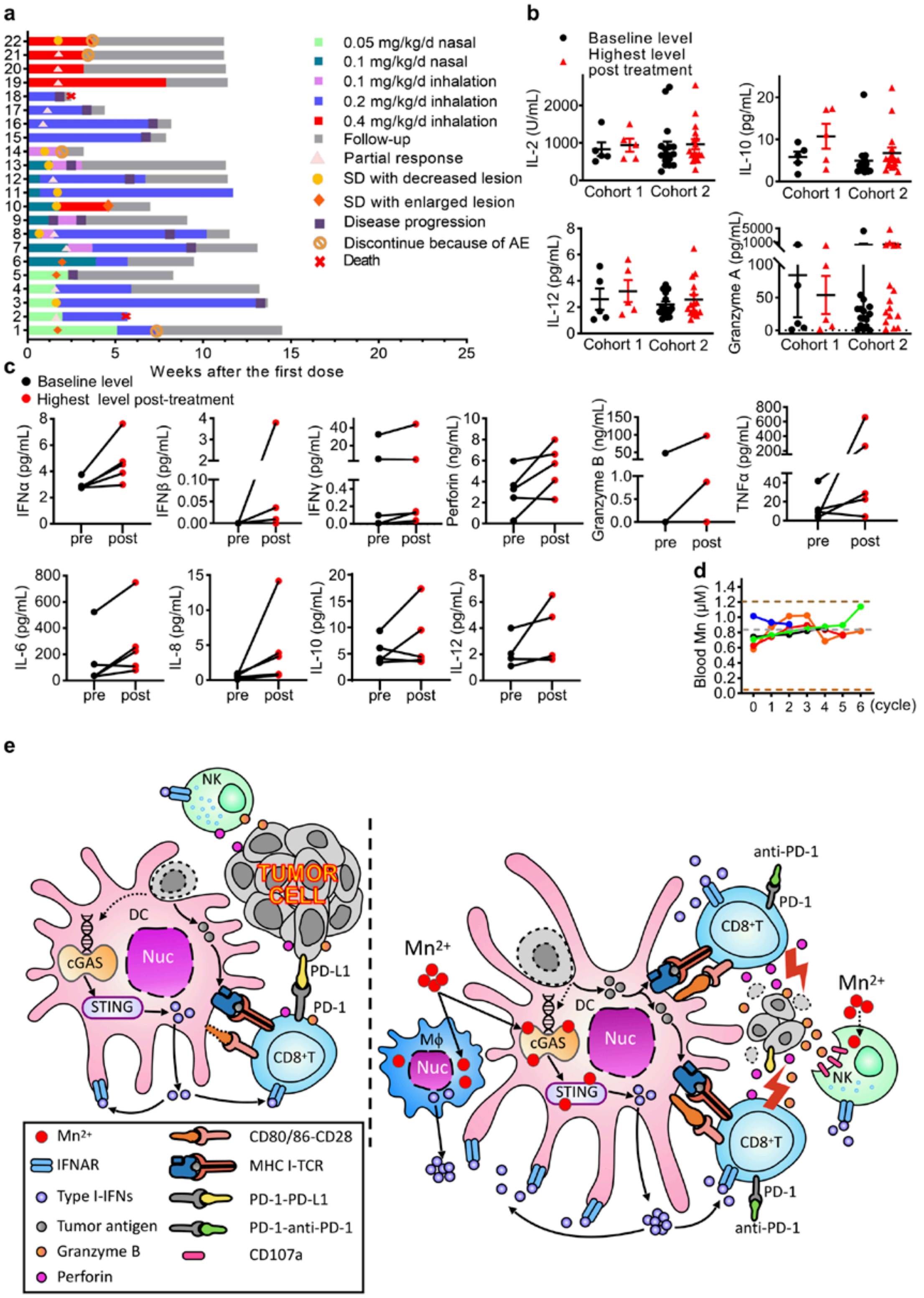


**Supplementary Fig. 7 $Mn^{2+}$ augments and revives antitumor immunotherapy in multidrug (immuno)-resistant cancer patients. a** The clinical course is illustrated in this swimmer plot, including the best response, on- or off-treatment status. **b** The baseline and highest post-treatment expression level of serum cytokines and effector proteins of patients grouped according to the blood manganese concentration. Cohort 1 includes the patients with SD-E and PD; cohort 2

includes patients achieving PR and SD-S. **c** The baseline and highest post-treatment expression level of serum cytokines and effector proteins of five metastatic patients with cytokine -release-syndrome (CRS). **d** The level of blood Mn concentration in each patient as in (**c**) one day before the numbered cycle of $Mn^{2+}$ administration and therapy (C1-C4). Dashed lines indicated Mn concentrations within the physiological range (top, 1.2 μM; bottom, 0.029 μM). **e** A working model for $Mn^{2+}$ augmented and revived antitumor immunotherapy. In normal conditions, NK cells exerts tumor immunosurveillance, also tumor antigen is taken by DCs which results in $CD8^{+}$ T cell priming and activation. However, in cancer patients, there is not enough tumor clearance presumably due to inadequate activation of NK cells and $CD8^{+}$ T cells because of the insufficient activation and maturation of antigen-presenting cells (APCs, macrophages, DCs etc.). $Mn^{2+}$ sensitizes and activates the cGAS-STING pathway in various cells including macrophages and DCs to produce type I-IFNs that promote not only the function of NK cells, but also APC maturation and antigen presentation, leading to $CD8^{+}$ T cell activation. Thus, $Mn^{2+}$ administration promotes anti-tumor immune responses by activating both $CD8^{+}$ T cells and NK cells for the clearance of $CD8^{+}$ T cell-sensitive and $CD8^{+}$ T cell-resistant tumors.
