# Supplementary material for: Manganese is critical for antitumor immune responses via cGAS-STING and improves the efficacy of clinical immunotherapy
Source: Cell Res. 2020 Aug 24;30(11):966–79. doi: 10.1038/s41422-020-00395-4 (PMC7785004; doi:10.1038/s41422-020-00395-4)
Supplement: Supplementary file 9 — Supplementary information, Fig. S8 [file 41422_2020_395_MOESM9_ESM.pdf]

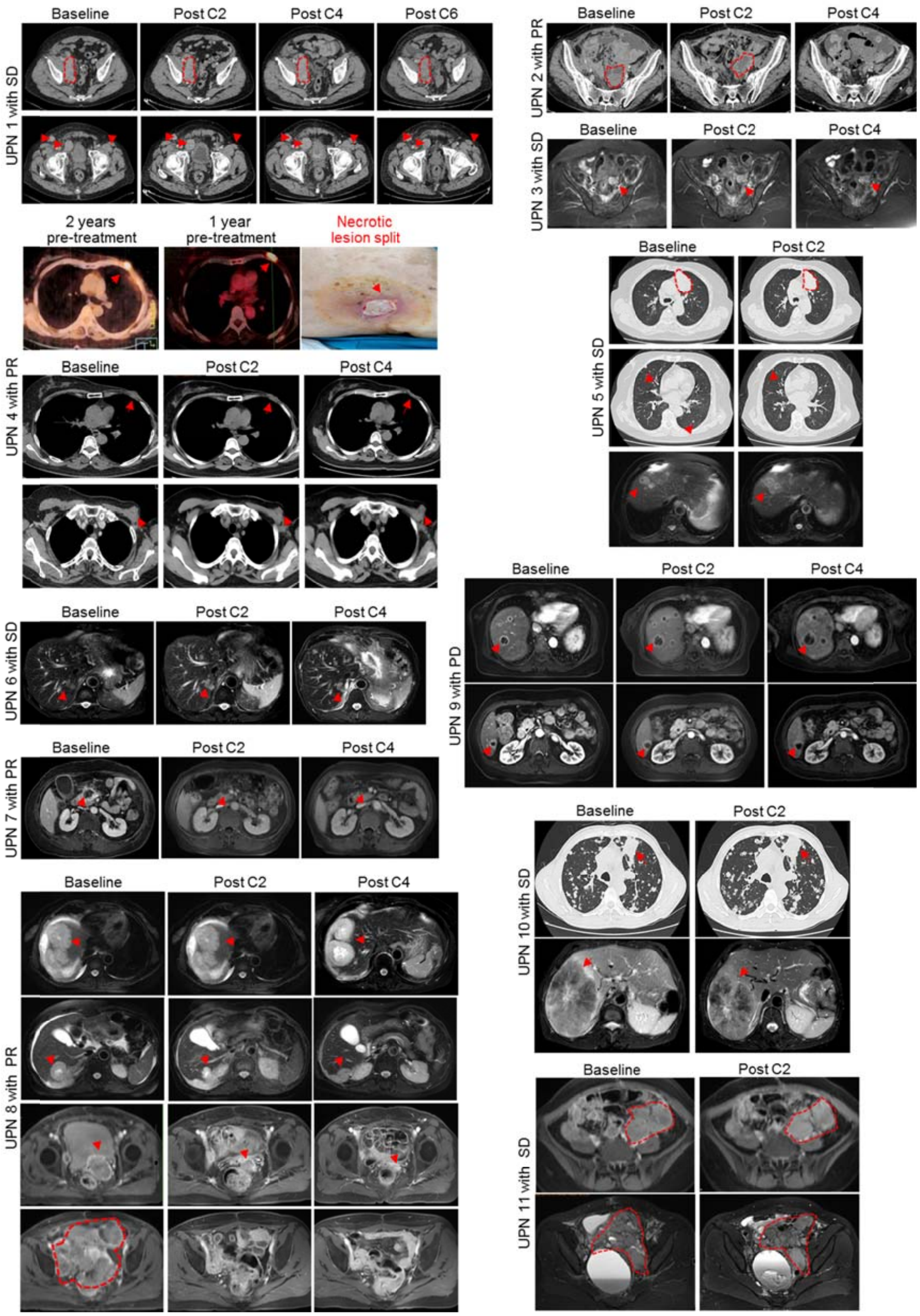

Baseline
Post C2
Post C4
Post C6
UPN 1 with SD
Baseline
Post C2
Post C4
UPN 2 with PR
Baseline
Post C2
Post C4
UPN 3 with SD
2 years pre-treatment
1 year pre-treatment
Necrotic lesion split
Baseline
Post C2
Post C4
UPN 4 with PR
Baseline
Post C2
UPN 5 with SD
Baseline
Post C2
Post C4
UPN 6 with SD
Baseline
Post C2
Post C4
UPN 9 with PD
Baseline
Post C2
Post C4
UPN 7 with PR
Baseline
Post C2
Post C4
UPN 8 with PR
Baseline
Post C2
UPN 10 with SD
Baseline
Post C2
UPN 11 with SD

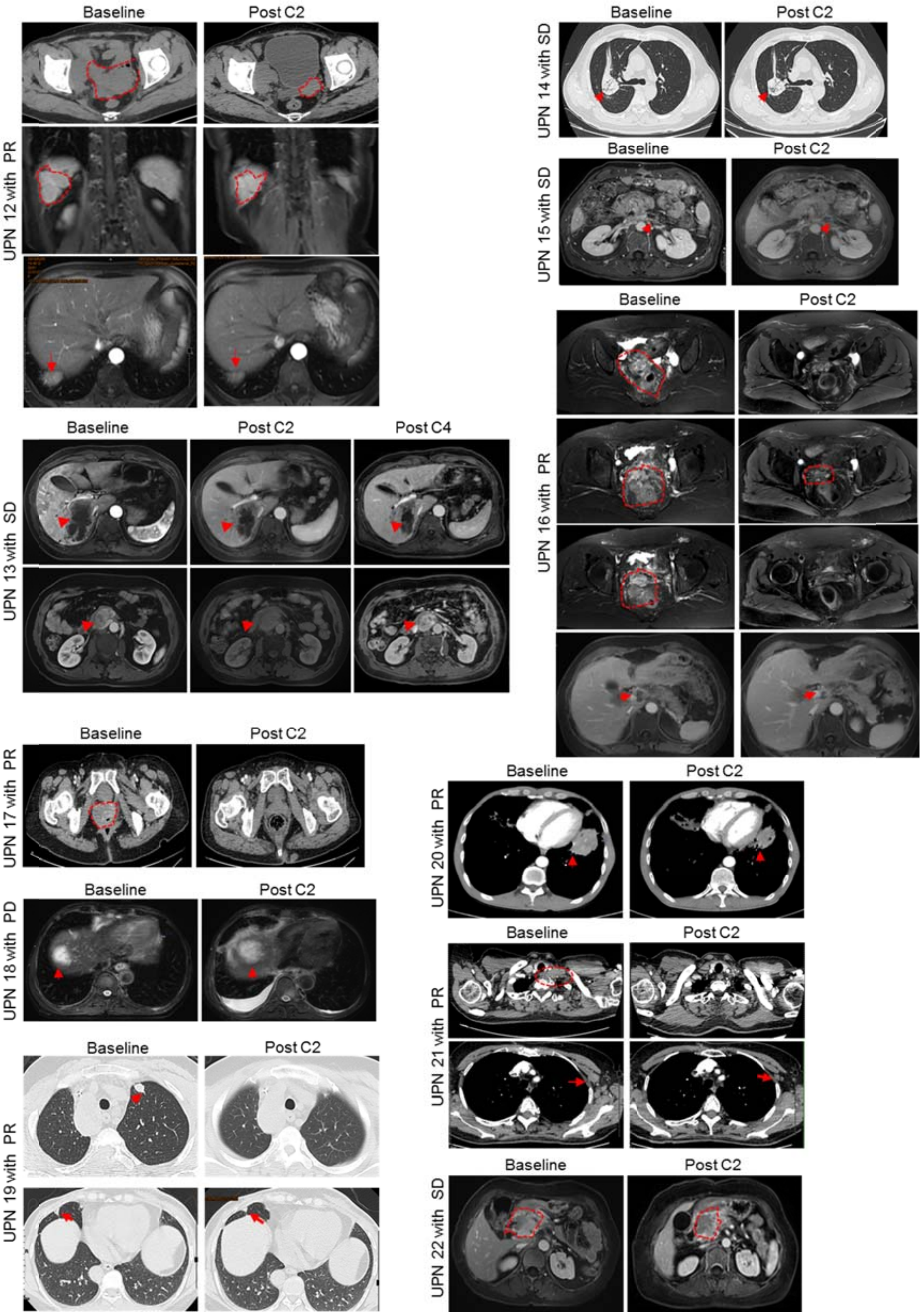

Supplementary Fig. 8 CT or MRI scans of all evaluated 22 patients. Computed tomography (CT) and/or magnetic resonance imaging (MRI) were performed at baseline and after every 2 cycles of the combined treatment.
