# Supplementary material for: Manganese is critical for antitumor immune responses via cGAS-STING and improves the efficacy of clinical immunotherapy
Source: Cell Res. 2020 Aug 24;30(11):966–79. doi: 10.1038/s41422-020-00395-4 (PMC7785004; doi:10.1038/s41422-020-00395-4)
Supplement: Supplementary file 10 — Supplementary information, Table S1 [file 41422_2020_395_MOESM10_ESM.pdf]

Supplementary Table 1. Quantitative PCR primers

mIfnb1	CACAGCCCTCTCCATCAACT	TCCCACGTCAATCTTTCCTC
mTnfa	GGGTGATCGGTCCCCAAAGG	CTCCACTTGGTGGTTTGCTACGA
Gapdh	TGATGGGTGTGAACCACGAG	TAGGGCCTCTCTTGCTCAGT
