# Supplementary material for: Manganese is critical for antitumor immune responses via cGAS-STING and improves the efficacy of clinical immunotherapy
Source: Cell Res. 2020 Aug 24;30(11):966–79. doi: 10.1038/s41422-020-00395-4 (PMC7785004; doi:10.1038/s41422-020-00395-4)
Supplement: Supplementary file 11 — Supplementary information, Table S2 [file 41422_2020_395_MOESM11_ESM.pdf]

Supplementary Table 2. Information of peripheral blood mononuclear cell donors, Related to Figure S4 and Table S4-S5

Patient Number	Age	Gender	Types of cancer	Mn^{2+} Response *in vitro*	Clinical response
1	46	Female	Pancreatic cancer	Yes	Not Enrolled
2	59	Female	Rectal cancer	Yes	Not Enrolled
3	55	Female	Pancreatic cancer	Yes	Not Enrolled
4	67	Female	Breast cancer	Yes	Not Enrolled
5	75	Male	Kidney cancer	Yes	Not Enrolled
6	44	Male	Pancreatic cancer	Yes	Not Enrolled
7	73	Female	Squamous Cell Carcinoma - Base of Tongue	Yes	Not Enrolled
8 (UPN13)	41	Male	Cholangiocarcinoma	Yes	SD-S
9	60	Male	Pancreatic cancer	Yes	Not Enrolled
10	61	Male	Cancer of the gastric cardia	No	Not Enrolled
11 (UPN9)	50	Female	Cholangiocarcinoma	Yes	PD
12 (UPN8)	58	Female	Fallopian tube cancer	Yes	PR
13 (UPN7)	63	Female	Pancreatic cancer	Yes	PR
14	36	Female	Breast cancer	Yes	Not Enrolled
15	40	Male	Floor of mouth cancer	Yes	Not Enrolled
16	50	Female	Colon cancer	No	Not Enrolled
17	47	Male	Gastric cancer	Yes	Not Enrolled
18 (UPN18)	69	Male	Pancreatic cancer	Yes	PD
19 (UPN12)	64	Female	Ovarian cancer	Yes	PR
20	54	Male	Gastric cancer	Yes	Not Enrolled
21	57	Male	Pancreatic cancer	Yes	Not Enrolled
22 (UPN21)	58	Female	Fallopian tube cancer	Yes	PR
23 (UPN19)	47	Female	breast cancer	Yes	PR
24 (UPN4)	55	Female	breast cancer	Yes	PR
25 (UPN20)	55	Male	Squamous cell lung carcinoma	Yes	PR
26 (UPN22)	62	Female	Pancreatic cancer	Yes	SD-S
27	62	Female	Pancreatic cancer	No	Not Enrolled
28	53	Female	Colon cancer	Yes	Not Enrolled
29	58	Female	Breast cancer	Yes	Not Enrolled
30	71	Male	Esophagus cancer	Yes	Not Enrolled
31	55	Male	Colon cancer	Yes	SD-S
32	62	Female	Peritoneal carcinomatosis	Yes	Not Enrolled
33	39	Female	Ovarian cancer	Yes	Not Enrolled
34	64	Female	Ovarian cancer	No	Not Enrolled
35	45	Female	Breast cancer	Yes	Not Enrolled

36	62	Male	Esophagus cancer	Yes	Not Enrolled
37	52	Female	Squamous cell thymic carcinoma	Yes	Not Enrolled
38 (UPN11)	30	Female	Ovarian cancer	Yes	SD-S
39 (UPN15)	41	Male	Pancreatic cancer	No	SD-S
40 (UPN16)	55	Female	Ovarian cancer	Yes	PR
41	44	Male	Pancreatic cancer	Yes	Not Enrolled
42	48	Female	Breast cancer	Yes	Not Enrolled
43	50	Female	Pancreatic cancer	Yes	Not Enrolled
44 (UPN3)	57	Female	Ovarian cancer	Yes	SD-S
45	27	Male	Cholangiocarcinoma	Yes	Not Enrolled
46	67	Female	Lung cancer	Yes	Not Enrolled
47	65	Male	Colon cancer	Yes	Not Enrolled
48	56	Male	gastric cancer	Yes	Not Enrolled
49 (UPN17)	71	Female	Ovarian cancer	Yes	PR
50 (UPN2)	56	Female	Fallopian tube cancer	Yes	PR
51 (UPN14)	49	Male	Lung Cancer	Yes	SD-E
