# Supplementary material for: Manganese is critical for antitumor immune responses via cGAS-STING and improves the efficacy of clinical immunotherapy
Source: Cell Res. 2020 Aug 24;30(11):966–79. doi: 10.1038/s41422-020-00395-4 (PMC7785004; doi:10.1038/s41422-020-00395-4)
Supplement: Supplementary file 13 — Supplementary information, Table S4 [file 41422_2020_395_MOESM13_ESM.pdf]

Supplementary Table 4. Baseline characteristics

Characteristics	All patients (n = 22)	Nasal drop (n = 13)	Inhalation* (n = 20)
Age, years			
Median	56	45	55.5
Range	30-78	30-78	30-72
Sex			
Female	14 (64%)	9 (69%)	11 (65%)
Male	8 (36%)	4 (31%)	7 (35%)
Cancer type			
Digestive system cancer	9 (41%)	6 (46%)	7 (35%)
Gynecological cancer	8 (36%)	5 (38%)	8 (40%)
Lung cancer	3 (14%)	1 (8%)	3 (15%)
Breast cancer	2 (9%)	1 (8%)	2 (10%)
Disease stage at enrollment			
IV	22 (100%)	13(100%)	20 (100%)
ECOG			
0	12 (54%)	7 (54%)	11 (55%)
1	7 (32%)	4 (31%)	6 (30%)
2	3 (14%)	2 (15%)	3 (15%)
Metastatic site			

Lymph node	20 (91%)	11 (85%)	20 (100%)
Liver	13 (59%)	10 (77%)	11 (55%)
Lung	6 (27%)	3 (23%)	4 (20%)
Bone	2 (9%)	1 (8%)	1 (5%)
Previous lines of therapy			
Median	3	3	3
Range	0-17	0-7	0-17
≥ 3 lines	13 (59%)	9 (69%)	12 (60%)
Previous surgery	17 (77%)	11 (85%)	15 (75%)
Previous anti-PD-1 antibody	6 (27%)	4 (31%)	6 (30%)

* This cohort contains eleven patients who were transferred from the nasal drop cohort (Cohort 1).
