# Supplementary material for: Manganese is critical for antitumor immune responses via cGAS-STING and improves the efficacy of clinical immunotherapy
Source: Cell Res. 2020 Aug 24;30(11):966–79. doi: 10.1038/s41422-020-00395-4 (PMC7785004; doi:10.1038/s41422-020-00395-4)
Supplement: Supplementary file 14 — Supplementary information, Table S5 [file 41422_2020_395_MOESM14_ESM.pdf]

Supplementary Table 5. The detailed baseline characteristics and enrolled treatment of all patients in the study

Patient No.	Age (years)	Sex	Cancer type	Enrolled stage	ECOG	Metastatic site	Previous treatment	Enrolled treatment
UPN1	72	M	CRC	IV	0	Inguinal LN; pelvic LN	Surgery, BEV+FOLFOX×4, BEV+FOLFIRI×4, FOLFIRNOX+anti-PD1×1, BEV+anti-PD-1×2	N1×5, A2×3; Bev×8; anti-PD-1 (Nivolumab)×8
UPN2	56	F	OC	IV	2	Pelvic metastasis lesion	Primary debulking surgery, P C×10, Secondary debulking surgery, DTC×5, (BEV+GP)×4, (anti-PD-1+PC)×4, (AC+anti-PD-1)×1, (nPC+anti-PD-1)×1	N1×2, A2×5; nPC×7; anti-PD-1 (Toripalimab)×7
UPN3	57	F	OC	IV	1	Pelvic metastasis lesion	Primary debulking surgery+PC×2, Secondary debulking surgery+PC×5, anti-PD1×2, (nPC+anti-PD-1×8, nPC×4	N1×2, A2×12; nPC×14; anti-PD-1 (Toripalimab)×14
UPN4	55	F	BC	IV	0	Armpit LN; Left chest wall metastases	Modified radical mastectomy, AC×8, navelbine+lobaplatin×2, GP×2, decitabine+anti-PD-1×4, Bev+AC×6, Surgery+radiotherapy, Bev+Liposomal Doxorubicin+Carboplatin×1	N1×2; A2×4; nP×6; anti-PD-1 (Toripalimab)×6
UPN5	78	F	CRC	IV	0	Lung; liver	Surgery, radioactive seed implantation (pulmonary metastasis), regorafenib×2, TACE (Liver metastases)	N1×3; Bev×3; anti-PD-1 (Nivolumab)×3
UPN6	56	M	HCC	IV	1	liver	Surgery, TACE×2, Cryocare Surgical System, Sorafenib, Lenvatinib	N2×5; A2×2; Lenvatinib×7; anti-PD-1 (Nivolumab)×7

UPN7	63	F	PC	IV	0	Pancreatic lesions	/	N2×2, A1×2, A2×4; nPG×8; anti-PD1 (Nivolumab)×8
UPN8	58	F	OC	IV	2	Pelvic metastasis lesion	Primary debulking surgery, PC×6, Olaparib maintenance, PC×2+Secondary debulking surgery, nPC×2	N2×1, A1×1, A2×8; nPC×10; anti-PD-1 (Pembrolizumab)×10
UPN9	50	F	CCA	IV	0	Liver	Surgery, GMOX×2	N2×2, A1×2; GP×4; anti-PD1 (Nivolumab)×4
UPN10	54	M	CRC	IV	1	Lung; liver	Surgery, Xelox×4, CET+FOLFIRI×4, CET×5	N2×2, A3×3; nP×5 anti-PD1 (Nivolumab)×5
UPN11	30	F	OC	IV	1	Pelvic metastasis lesion	Primary debulking surgery+(Liposomal Paclitaxel +Carboplatin)×8, Liposomal Paclitaxel ×3, (Liposomal Doxorubicin+lobaplatin) ×2	N2×1, A2×13; nPC×14; anti-PD-1 (Pembrolizumab)×14
UPN12	64	F	OC	IV	0	Pelvic metastasis lesion	PC×1+Interval debulking surgery+(P+lobaplatin)×7, PC×3, (nab-PTX+lobaplatin)×1	N2×1, A2×6; nPC×7; anti-PD-1 (Nivolumab)×7
UPN13	41	M	CCA	IV	0	Liver; retroperitoneal LN	TACE×1	N2×3, A1×3; GP×6; anti-PD-1 (Nivolumab)×6
UPN14	49	M	LC	IV	1	Lung	Bev+AP×4, Bev+AP+SHR-1210×2, Bev+A+SHR-1210×14, Bev+nab-PTX+DDP×6	A1×1, A2×2; nP×3; anti-PD-1 (SHR-1210)×3
UPN15	41	M	PC	IV	0	Retroperitoneal LN	Surgery, radiotherapy	A2×6; GP×6; anti-PD-1 (Nivolumab)×6
UPN16	55	F	OC	IV	0	Pelvic metastasis lesion	Primary debulking surgery, PC×7, Apatinib, Liposomal Doxorubicin×3, (Docetaxel+Cisplatin)×2, Bev+G×6	A2×8; nPC×8; anti-PD-1 (Pembrolizumab)×8

UPN17	71	F	OC	IV	2	Pelvic metastasis lesion	Surgery×4, chemotherapy×35, anti-PD-1×8, Niraparib, Olaparib	A2×4; nPC×4; anti-PD-1 (Pembrolizumab)×4
UPN18	69	M	PC	IV	1	Liver; retroperitoneal LN	Surgery, GPx6, S1+ErotinibX1	A2×3; FOLFIRINOX×1; Lenvatinib×2; anti-PD-1 (Sintilimab)×3
UPN19	47	F	BC	IV	0	Lung; supraclavicular LN	Modified radical mastectomy (Right)+AC-T×3, Radiotherapy, Modified radical mastectomy (Left)	A3×9; nP×9; anti-PD-1 (Nivolumab)×9
UPN20	55	M	LC	IV	1	Lung	GP×5, AP×1	A3×4; nPT×4; anti-PD-1 (Pembrolizumab)×4
UPN21	58	F	OC	IV	0	Neck LN	Primary debulking surgery+PC×4, Secondary debulking surgery+PC×4	A3×4; nPC×4; anti-PD-1 (Sintilimab)×4
UPN22	62	F	PC	IV	0	Pancreatic lesions	/	A3×4; nPG×4; anti-PD-1 (Sintilimab)×4

BC, Breast cancer; CCA, Cholangiocarcinoma; CRC, Colorectal cancer; HCC, Hepatocellular carcinoma; LC, Lung cancer; OC, ovarian cancer; PC, Pancreatic Cancer. N1, N2 represent intranasal administration of manganese chloride 0.05 and 0.1 mg/Kg/d, respectively; A1, A2 and A3 represent inhalation of manganese chloride 0.1, 0.2 and 0.2 mg/Kg/d, respectively.
