# Supplementary material for: Manganese is critical for antitumor immune responses via cGAS-STING and improves the efficacy of clinical immunotherapy
Source: Cell Res. 2020 Aug 24;30(11):966–79. doi: 10.1038/s41422-020-00395-4 (PMC7785004; doi:10.1038/s41422-020-00395-4)
Supplement: Supplementary file 15 — Supplementary information, Table S6 [file 41422_2020_395_MOESM15_ESM.pdf]

Supplementary Table 6. Treatment-emergent adverse events

	Total patients						Cohort 1				Cohort 2			
	Any grade		Grade1-2		Grade3-4		Grade1-2		Grade3-4		Grade1-2		Grade3-4	
	No. of patients	%	No. of patients	%	No. of patients	%	No. of patients	%	No. of patients	%	No. of patients	%	No. of patients	%
Any adverse event	19	86	10	45	9	41	3	14	0	0	7	32	9	41
Peripheral neuropathy	14	64	14	64	0	0	1	5	0	0	13	59	0	0
Leucopenia	12	55	12	55	0	0	0	0	0	0	12	55	0	0
Nausea	8	37	8	37	0	0	0	0	0	0	8	37	0	0
Vomiting	8	37	5	23	3	14	0	0	0	0	5	23	3	14
Thrombocytopenia	7	32	5	23	2	9	0	0	0	0	5	23	2	9
Neutropenia	7	32	4	18	3	14	0	0	0	0	4	18	3	14
Fatigue	4	18	4	18	0	0	0	0	0	0	4	18	0	0
Constipation	4	18	4	18	0	0	1	5	0	0	3	14	0	0
Fever	4	18	4	18	0	0	1	5	0	0	3	14	0	0
Rash	3	14	3	14	0	0	0	0	0	0	3	14	0	0
Pruritus	3	14	3	14	0	0	0	0	0	0	3	14	0	0
Diarrhea	2	9	2	9	0	0	0	0	0	0	2	10	0	0
Ileus	2	9	2	9	0	0	1	5	0	0	1	5	0	0
Pneumonitis	2	9	0	0	2	9	0	0	0	0	0	0	2	9
Anorexia	1	5	1	5	0	0	0	0	0	0	1	5	0	0
Myalgia	1	5	1	5	0	0	0	0	0	0	1	5	0	0
Ascites	1	5	1	5	0	0	0	0	0	0	1	5	0	0
Hydrothorax	1	5	0	0	1	5	0	0	0	0	0	0	1	5
